# Targeting Adaptive and Innate Immune Responses with Covalent Aptamers

**DOI:** 10.1021/jacs.6c10475

**Published:** 2026-08-10

**Authors:** Savannah Albright, Avani Parikh, Mary Cacace, Carolyn Rosenblum, Michael Dorogan, Jason Lohmueller, Alexander Deiters

**Affiliations:** † Department of Chemistry, 6614 University of Pittsburgh, Pittsburgh, Pennsylvania 15260, United States; ‡ Department of Surgery, Division of Surgical Oncology, University of Pittsburgh, Pittsburgh, Pennsylvania 15213, United States; § Institute for Synthetic Biology, University of Pittsburgh, Pittsburgh, Pennsylvania 15260, United States

## Abstract

Covalent aptamers have recently found initial success as tools for fast and selective transfer of labels to target proteins. Here, aptamers targeting protein tyrosine kinase 7 (PTK7) and the mesenchymal-epithelial transition factor (cMet) were functionalized with electrophiles capable of covalently transferring small-molecule immune recruiters. Capitalizing on biotin transfer, aptamers were utilized to trigger activation of universal anti-biotin chimeric antigen receptor (CAR) T cells resulting in selective lysis of PTK7- and cMet-positive cells. Additionally, a cleavable electrophile of 2,4-dinitrophenyl (DNP) hapten transfer was synthesized and utilized for the selective modification of cell surface PTK7 and cMet. The DNP modification elicited the selective activation of complement-dependent cytotoxicity (CDC). These results demonstrate the potential of covalent aptamers as immune-activating and cancer-cell-killing agents.

## Graphical abstract

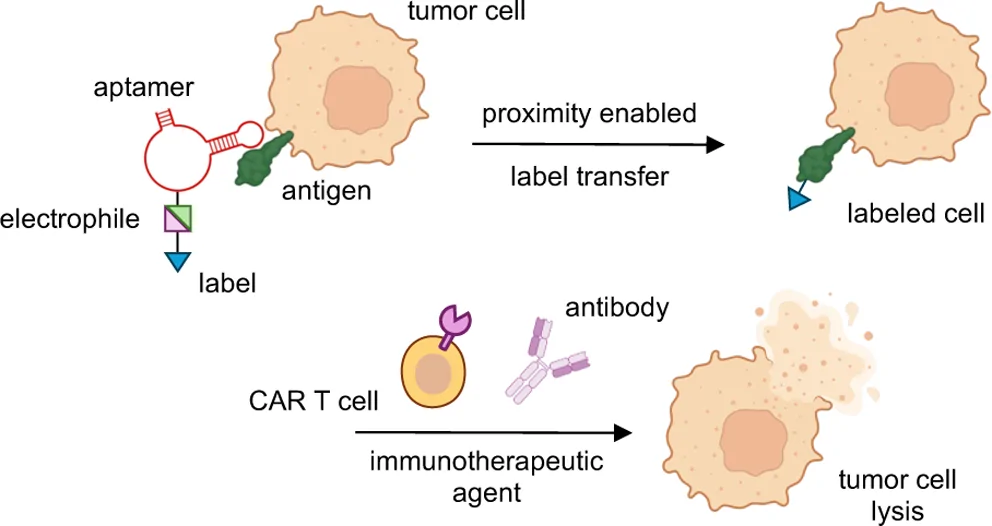

## Introduction

Immunotherapy has transformed the field of cancer treatment by harnessing the body’s own immune system to recognize and destroy tumor cells, offering the promise of more precise, durable, and potentially life-saving therapies.^1,2^ Immunotherapy approaches have shown remarkable efficacy, a prolonged therapeutic response, and decreased side effects.^3–6^ Several immunotherapeutics have achieved FDA approval, including checkpoint inhibitors,^7,8^ CAR T cells,^9^ and bispecific antibodies.^10^ These approaches can also function in tandem with more traditional chemotherapy and radiotherapy as a multimodal approach to target tumors,^11–13^ potentially allowing for lower dosage and frequency of cytotoxic agent treatment in order to facilitate improved survival.^14–16^ This wide-ranging field has revolutionized cancer treatment and is allowing for unique, personalized treatment of patients.

Chimeric antigen receptors (CARs)^17^ are synthetic receptors that have been engineered to redirect the specificity of patients’ T cells toward tumor associated antigens (TAAs).^18^ These CARs contain T cell signaling and costimulatory domains fused to an antigen recognition domain, usually a single-chain variable fragment, which directs the cells toward a cognate TAA.^19,20^ To date, seven CAR T cell therapies, targeting CD19 and BCMA to treat various blood cancers including B cell leukemias, lymphomas,^21–24^ and multiple myeloma,^25,26^ are FDA-approved. Despite the success of these therapies, CAR T cells face some inherent limitations, such as high cost of production, therapeutic resistance due to antigen loss, various toxicities from on-target off-tumor effects, and overactivation of immune cells such as cytokine release syndrome.^27,28^

To address the limitations of traditional CARs, universal CAR T cells have been developed. While traditional CARs recognize a specific TAA, this next generation of CAR T cells express a “universal” receptor that binds to TAA specific “adaptor” molecules, targeting the CAR T cell to one or more antigen(s).^29^ Examples of previously established CAR-binding motifs used on adaptors include FITC,^30^ peptide-neoepitopes,^31^ leucine zippers,^32^ biotin,^33^ benzylguanine,^34^ and SpyTag.^35^ While universal CAR T cells have been activated through either covalent or noncovalent binding to the adaptor molecule, here we aim to directly mark target cells with the adaptor tag molecule via covalent aptamers.

Aptamers are short, single-stranded RNA or DNA oligonucleotides with high specificity and affinity for their target proteins. Aptamers have distinct advantages over small molecules as protein-targeting ligands: the ability to bind to proteins that do not have a distinct small molecule-binding pocket,^36^ their discovery through selection processes both in vitro and in vivo, and their general amenability to modification with electrophiles due to their lack of nucleophilic moieties resembling amino acid side-chains.^37,38^ While the concept of covalent aptamers was first reported in 1995,^39^ covalent aptamers have received increasing attention in recent years.^40,41^

Here, we are capitalizing on our development of covalent aptamers capable of label transfer to their target through the use of a cleavable *N*-acyl, *N*-alkyl sulfonamide electrophile to achieve a targeted immune response that destroys cancer cells (Figure 1A) through two different approaches: activation of engineered T cells and activation of a complement response. First, we use the biotinylation of cell surface biomarkers to recruit affinity-enhanced monomeric streptavidin 2 (mSA2)-CAR T cells and induce a cytotoxic response.^42^ Previously, biotinylated antibodies have been generated that bind TAAs and recruit and subsequently activate mSA2-CAR T cells, however, covalent bond formation to the TAA has not been reported. This would be difficult to achieve with antibodies due to the presence of nucleophilic amino acids on their surface—including over 60 lysine residues;^43^ however, select examples of installation of electrophilic groups via unnatural amino acid mutagenesis have been reported.^44–46^ In contrast, site-selectively electrophile-modified aptamers can be readily synthesized, are stable, and less expensive than antibodies.

**Figure 1:**
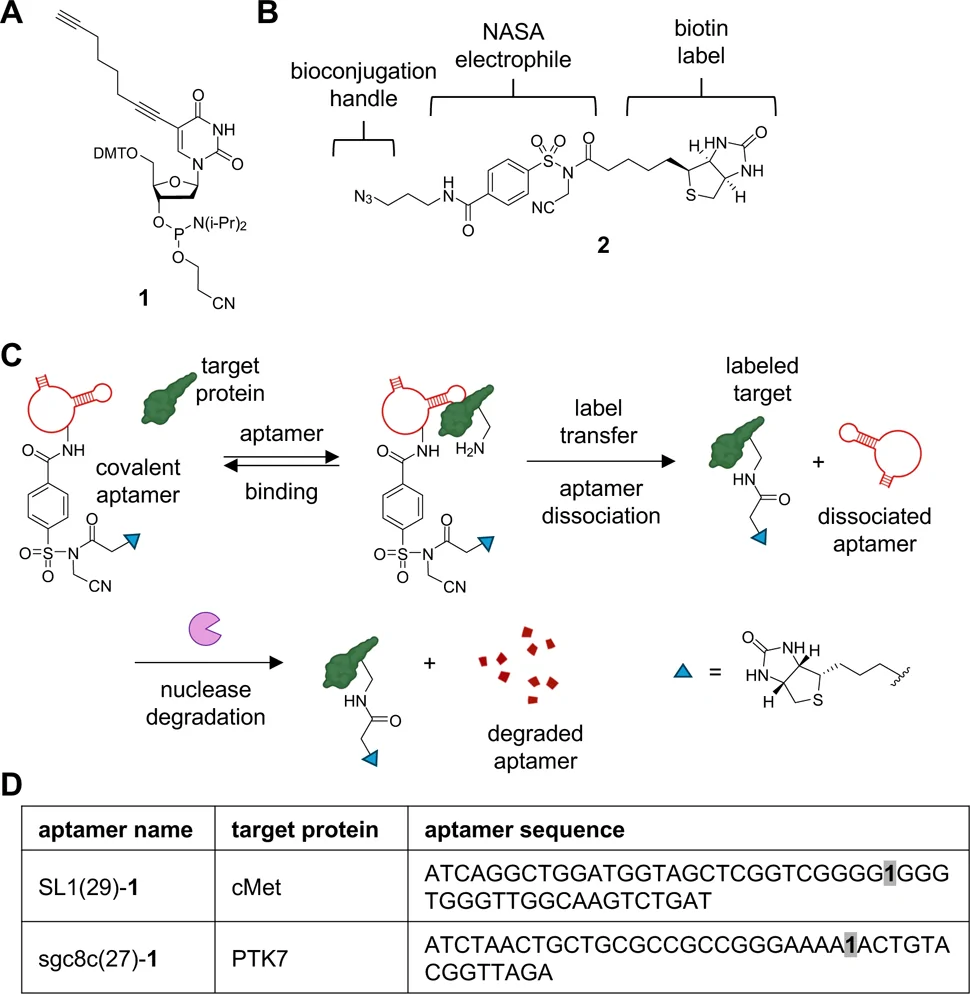
(A) Structure of modified 5-octadiynyl-2′-deoxyuridine (**1**). (B) Structure of biotinylating warhead **2**. (C) Cartoon schematic and mechanism of aptamer-mediated protein biomarker labeling and subsequent aptamer dissociation and degradation. (D) Sequences of aptamers used in this study. Optimal warhead position was identified by SAR (modified residue is bolded and highlighted). A full list of aptamer sequences used in this study can be found in Supporting Information Table S2.

## Results and Discussion

To expand upon our recently developed covalent aptamers targeting thrombin and PTK7,^40,41^ an aptamer targeting the mesenchymal-epithelial transition factor (cMet) was functionalized by site-selective modification of the DNA aptamer SL1.^47,48^ The receptor tyrosine kinase cMet contains four extracellular immunoglobulin domains and plays a key role in tissue homeostasis, embryogenesis, and wound healing.^49^ Dysregulation and overactivation of cMet drive oncogenesis and result in poor prognosis in various cancers, including gastric,^50^ colorectal,^51^ lung,^52^ and breast.^53^ SL1, which has been predicted to fold into a G-quadruplex-containing stem-loop structure, has been shown to inhibit cMet signaling^48^ and cell migration.^54^

A full panel of covalent SL1 positional isomers was synthesized using 5-octadiynyl-2′-deoxyuridine phosphoramidite (OdU **1**, Figure 1A) in place of individual thymidine residues. The biotin-delivering, ligand-directed NASA electrophile **2** (Figure 1B) was installed through Cu-catalyzed [3 + 2] cycloaddition.^41^ Upon target binding, nucleophilic attack of the sulfonamide by a lysine results in covalent transfer of the biotin to the protein, permitting aptamer dissociation and subsequent degradation by endogenous nucleases (Figure 1C). The sequences of the modified aptamers used in this study are reported in Figure 1D (all sequences in Supporting Information Table S2).

With the synthesized panel of covalent aptamers in hand, a structure-activity relationship (SAR) study was performed to identify the position that afforded the most efficient labeling of recombinant cMet., establishing positions 8, 15, 23, and 29 as functional (Figure 2A). However, when the same SAR study was performed on cells expressing cMet, only positions 15 and 29 successfully labeled cMet (Figure 2B, Supporting Information Figure S1). Position 29 was chosen for subsequent experiments, as it is located in the stem-loop portion of the aptamer and previous studies with our PTK7-labeling covalent aptamer showed that placement of the electrophile within the flexible loop portion provided optimal labeling efficiency.^41,55^ While the SARs for recombinant and cell-surface PTK7 were similar, differences observed for cMet likely reflect the distinct structural and environmental contexts of the receptor. At the cell surface, cMet is membrane-anchored, extensively post-translationally modified via glycosylation and ubiquitination, and capable of dimerization, which may constrain receptor orientation and reduce accessibility of surface lysine residues to certain aptamer positions, such as position 23, compared to the more flexible soluble protein.^49,56–61^ Consistent with this, modeling suggests that position 29, located within the flexible loop region of the SL1 aptamer, might be better oriented for electrophile transfer than position 23, limiting proper orientation in the more constrained cell-surface environment (Supporting Information Figure S2).^62^

**Figure 2:**
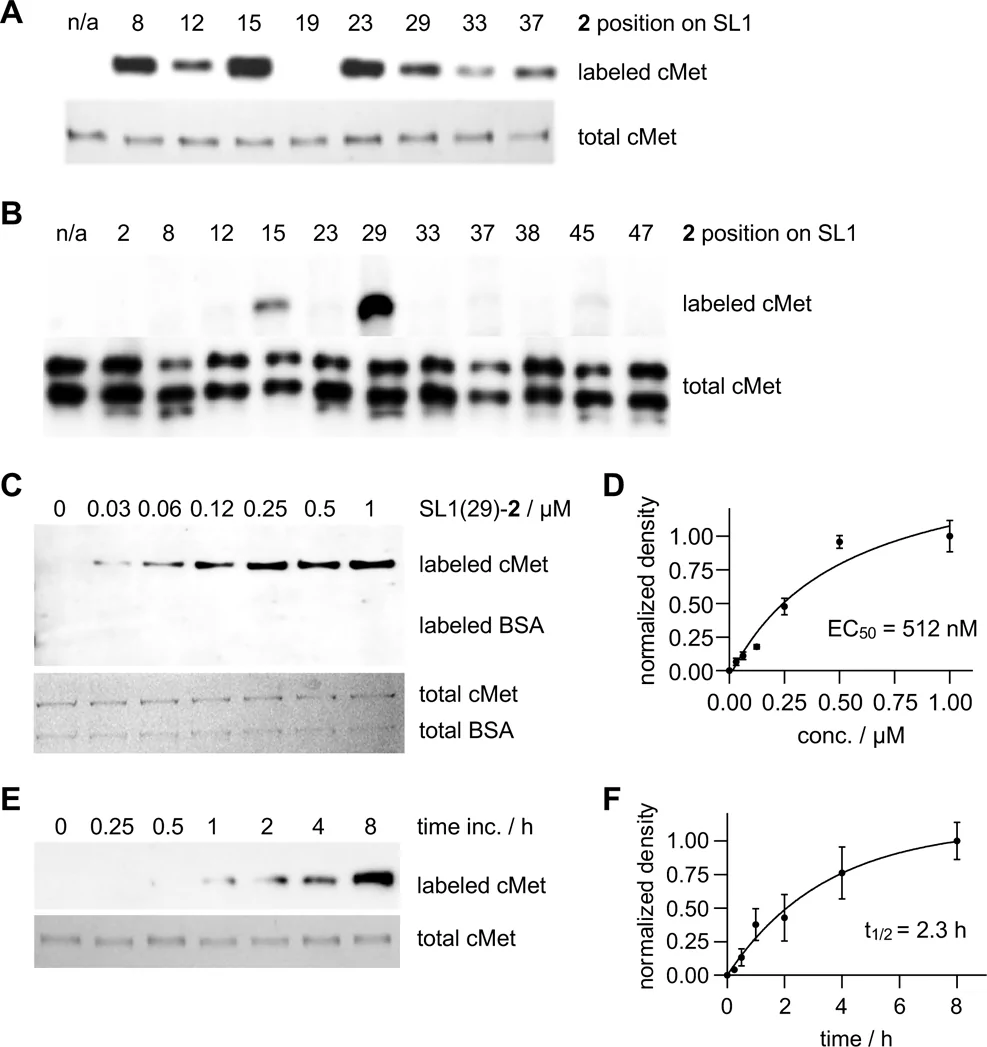
(A) SAR analysis of SL1 (0.25 μM) labeling cMet (0.1 μM) with the warhead **2** (SL1-**2**). (B) SAR analysis of SL1-**2** labeling cMet on transfected HEK293T cells. The two bands of cMet are a result of the mature β-subunit (145 kDa) and the single-chain precursor (175 kDa).^63,64^ (C) Selectivity analysis of SL1(29)-**2** incubated with 0.1 μM of both cMet and BSA. (D) Quantification of SL1(29)-**2** titration to determine the EC_50_ of labeling. (E) Representative blot of time-dependent labeling of cMet (0.1 μM) via SL1(29)-**2** (0.25 μM). (F) Quantification of time-course labeling to determine *t*_1/2_. Biotinylation was visualized by SA-HRP blotting. Total protein was visualized either by silver stain (recombinant) or anti-GFP blot (cell-surface). Data points represent averages and error bars represent standard deviation of triplicate data.

Dose titration of SL1(29)-**2** in the presence of a 1:1 mixture of cMet and BSA, as an off-target control, showed specific biotinylation of cMet (Figure 2C). BSA was chosen due to its presence in serum and its abundance in lysine residues (59 total). Quantification of triplicate data revealed an EC_50_ of 512 nM (95% CI: 0.15–7.86) (Figure 2D, Supporting Information Figure S3), which is comparable, though slightly higher, than the *K*_D_ of SL1 for recombinant cMet (237 nM).^48^ The slight decrease in affinity could be a result of warhead installation. A time course experiment using a 0.25 μM concentration of SL1(29)-**2** to label recombinant cMet (0.1 μM) demonstrated a *t*_1/2_ of 2.3 h (Figure 2E,F, Supporting Information Figure S4), slightly longer than what was observed for labeling of PTK7 and thrombin with label-transferring aptamers.^40,41^ Although the apparent *t*_1/2_ of labeling of cMet was slightly longer than that observed for PTK7 labeling,^41^ comparison of the nonlinear regression fits indicated that the decay constants were not statistically different (extra sum-of-squares F test, *p* = 0.1533).

The cMet-targeting covalent aptamer was then utilized for delivery of biotin to cMet expressed on transfected HEK293T cells. First, the kinetics of biotin transfer to cell-surface cMet-GFP were evaluated. When translating to a cellular environment, in hopes of decreasing the amount of time necessary to reach a plateau of biotin transfer, the concentration of aptamer was increased to 0.5 μM, which indeed decreased the *t*_1/2_ of surface labeling to approximately 1 h without affecting specificity of labeling (Figure 3A, Supporting Information Figure S5). At a 0.5 μM concentration of SL1(29)-**2**, the *t*_1/2_ of cell surface labeling was approximately 1 h (Figure 3C), thereby favorably competing with nuclease-mediated degradation of aptamers in cell-based applications,^41^ which was previously determined for sgc8c to be ∼2 h and SL1 has shown stability up to 72 h, both in serum.^41,48^ The hour-scale labeling kinetics observed here are consistent with ligand-directed covalent labeling systems employing moderately reactive electrophiles, in which target occupancy is expected to be saturated and the covalent bond-forming step becomes rate-limiting to preserve labeling selectivity and minimize nonspecific reactivity.^65–67^ As a proof-of-concept for avidin binding to biotinylated cells, conditions for fluorescent readout of a tetramethylrhodamine-labeled NeutrAvidin (NA-TAMRA) construct were optimized (Figure 3A).

**Figure 3:**
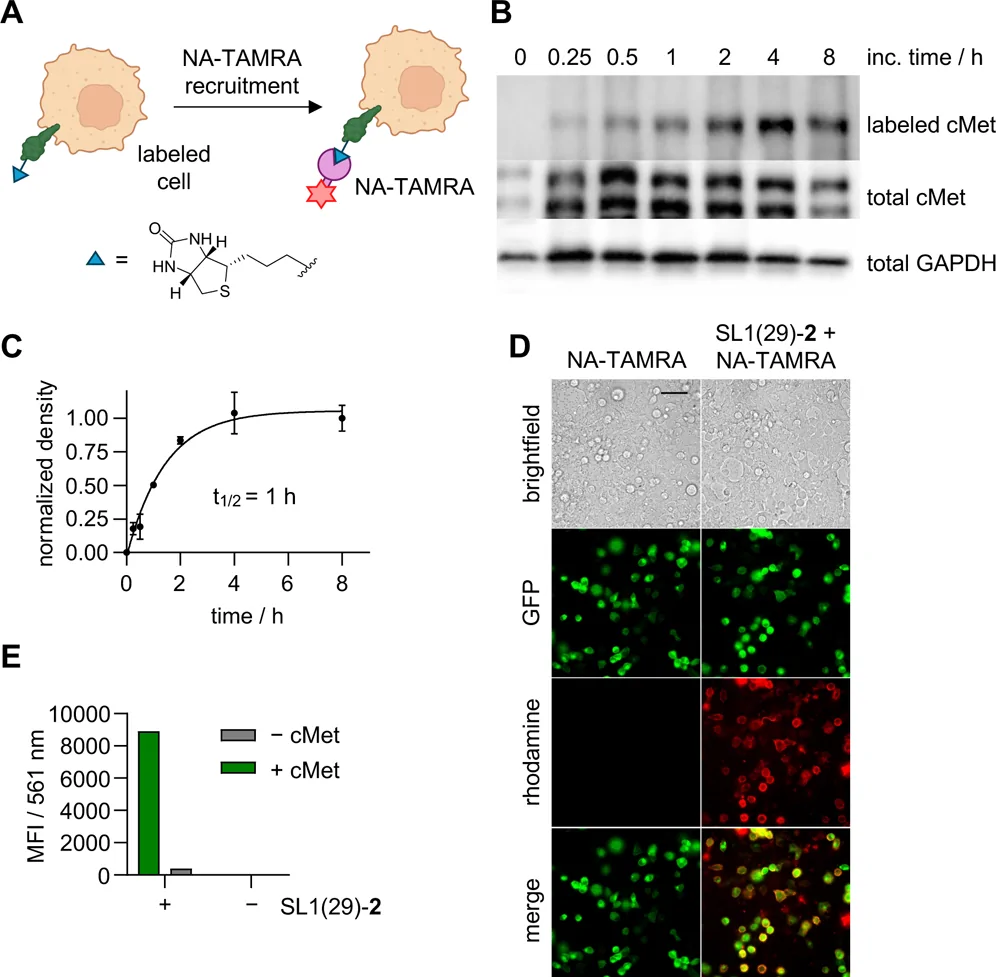
(A) Schematic depicting NeutrAvidin-TAMRA (NA-TAMRA) association of biotin-labeled tumor cells. (B) Representative blot and (C) quantification of time-dependent biotin transfer to cMet-expressing HEK293T cells by SL1(29)-**2** (0.5 μM). Biotinylation was analyzed via SA-HRP blot and total protein was visualized via anti-GFP blot. Data points represent averages and error bars represent standard deviations of three independent experiments. (D) Fluorescence imaging analysis of cMet positive and negative cells labeled with SL1(29)-**2** (0.5 μM) for 2 h, followed by NA-TAMRA incubation for 5 min. Scale bar = 50 μm. (E) Mean fluorescence intensity (MFI) of flow cytometry analysis of cells labeled as described in (D). 20,000 events were recorded.

To visualize the localization of the biotin modification of cMet at the cell surface, transfected HEK293T cells were incubated with 0.5 μM of SL1(29)-**2** for 2 h, at which point the reaction plateaued (Figure 3C). Cells were then incubated with NA-TAMRA and imaged.^41^ Excellent labeling specificity was observed, as only cMet-expressing cells (as indicated by a GFP fusion) showed red NA-TAMRA fluorescence (Figure 3D, Supporting Information Figure S6). Quantitative colocalization analysis revealed strong overlap between the rhodamine (biotin) and GFP (cMet) signals, with a Pearson’s coefficient of 0.85, indicating highly correlated spatial localization. By extending the incubation time after aptamer-mediated biotinylation before probing with NA-TAMRA, cell-surface biotinylation could still be observed after 16 h (Supporting Information Figure S7), suggesting sufficient duration of signal for use for further biological application. Additionally, NA-TAMRA recruitment was analyzed via flow cytometry and a 22-fold increase in mean red fluorescence intensity of cMet-GFP positive cells compared to the cMet-negative HEK293T cells in the same, mixed population (Figure 3E, Supporting Information Figure S8). Taken together, fast and selective labeling of cMet was achieved in a human cell setting.

Protein tyrosine kinase 7 (PTK7) is a biomarker for colon,^68^ nonsmall-cell lung,^69^ gastric,^70^ and cervical cancers;^71^ has been implicated in tumorigenesis;^72^ and—not surprisingly—is a target of various immunotherapies, including traditional CAR-T cells^70^ and antibody-drug conjugates (ADCs).^73^ To target PTK7 for aptamer-mediated immune recruitment, our previously established sgc8c(27)-**2** aptamer was first validated for efficient biotinylation of a stable HEK293T cell line expressing PTK7 through time-course-based labeling, as well as fluorescent NA-TAMRA recruitment detection via flow cytometry and fluorescence imaging (Supporting Information Figure S9).^41^ Unsurprisingly, these results showcased that our covalent sgc8c aptamer retained specificity and affinity for PTK7 encoded in both a stably modified genome and a transiently delivered vector.

With selective aptamer-mediated biotinylation of both cMet- and PTK7-expressing human cells confirmed, we next tested the ability to recruit mSA2-CAR T cells for targeted cell lysis (Figure 4A, Supporting Information Figure S10). In contrast to the generation of universal CAR adaptors through biotinylation of antibodies, the covalent aptamers directly biotinylate the target cell-surface-expressed TAA, forgoing the need for antibody modification and treatment.^42^ In order to test this, sgc8c(27)-**2** was added to HEK/PTK7-GFP cells. Cells were incubated for 2 h to achieve maximum label transfer, washed to remove excess aptamer, and stained with Cell Trace Yellow. The target cells were then incubated with mSA2-CAR T cells for 48 h to allow sufficient time for T cell activation and lysis to occur. Activation of T-cell signaling was measured via upregulation of CD69 expression.

**Figure 4:**
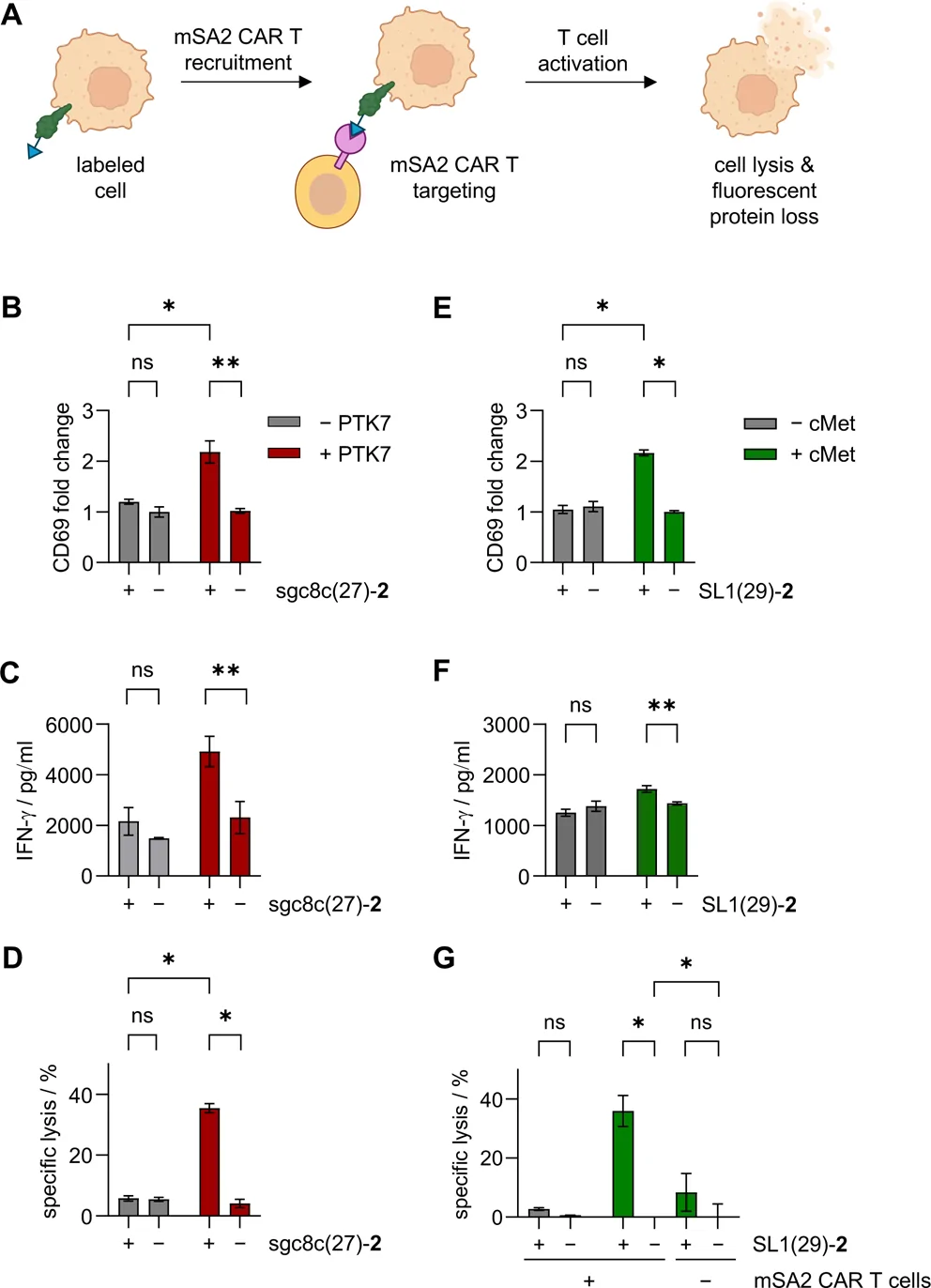
(A) mSA2-CAR T cell recruitment, activation, and target cell lysis in response to covalently biotinylated cancer biomarkers. (B) Mean fluorescence intensity (MFI) of CD69 expression and (C) IFN- γ production of mSA2-CAR T cells coincubated with biotinylated PTK7-positive cells. (D) Specific lysis of biotinylated HEK/PTK7 cells. (E) MFI of CD69 expression and (F) IFN-γ production of mSA2-CAR T cells coincubated with biotinylated cMet-positive HEK293T cells. (G) Specific lysis of cells. ns = not significant. * = *p* < 0.05. ** = *p* < 0.005. For flow cytometry, 20,000 events were recorded. MFI of CD69 levels were normalized to antigen negative, nontreated cells. Bars represent averages and error bars represent standard deviations from three independent experiments.

Expression of CD69, as detected by flow cytometry, was upregulated only when mSA2-CAR T cells were coincubated with biotinylated, PTK7-positive HEK293T cells, compared to untreated cells and PTK7-negative HEK cells (Figure 4B, Supporting Information Figure S10). We observed significant increase in CD69 expression indicating that successful activation of CAR T cell signaling resulted from binding of biotinylated cells. Additionally, a significant increase in IFN-γ production by mSA2-CAR T cells was only observed upon coincubation with biotinylated target cells, further supporting that selective labeling of PTK7 by sgc8c(27)-**2** allows for activation of CAR T cell effector functions (Figure 4C). Gratifyingly, activation of mSA2-CAR T cells also resulted in robust specific lysis of PTK7-positive HEK cells (Figure 4D). The amount of specific lysis (35%) is consistent with lysis observed with scFv/antibody-directed universal and traditional CAR T cells.^42,70^ Target cell lysis was titratable by increasing effector to target cell ratios, confirming that the induced lysis results from CAR-dependent recognition of biotinylated surface proteins (Supporting Information Figure S10C).

While the PTK7-positive cells show homogeneous antigen expression due to genomic integration, the transient expression of cMet-GFP resulted in a mixed population of cMet positive and cMet negative cells. Regardless, aptamer-mediated biotinylation of surface cMet resulted in comparable activation of mSA2-CAR T cells as indicated by CD69 marker upregulation and slight, but significant, IFN-γ production selectively in the presence of biotinylated HEK293T cells (Figure 4E,F).

Lysis of cMet+ cells was determined as a function of the percentage of GFP-positive cells in the population as has been previously described (Figure 4G, Supporting Information Figure S10D).^74^ A significant decrease (∼35%) of GFP-expressing cells in the population of biotinylated cells coincubated with mSA2-CAR T cells was observed, with no significant change from the aptamer alone or mSA2-CAR T cells alone. There was no significant toxicity observed on off-target cells subjected to aptamer labeling and subsequently coincubation with mSA2-CAR T cells. Although a direct experimental comparison was not performed in this study, the efficacy of cytotoxicity observed here is comparable to that reported in previous antibody-based adaptor systems. In the original antibody-based mSA2-CAR report, the highest levels of target cell lysis achieved by mSA2-CAR T cells were approximately 25–35% against CD19- and CD20-expressing targets.^42^ Using the covalent aptamer-enabled tagging strategy described here, we observed a comparable cytotoxic response of ∼35%, albeit for different antigens. These results suggest that aptamer-mediated covalent receptor tagging can support CAR T-mediated killing at levels similar to those obtained using antibody-based adaptor strategies.

The results discussed above demonstrate the ability to utilize covalent aptamers for the marking of specific cells to be lysed by CAR T cells. In order to alleviate the need for T cell engineering and subsequent implantation into patients required for current CAR T cell therapeutics,^75–77^ we next set out to covalently modify cancer cells for engaging an endogenous immune response. Antibody recruiting molecules (ARMs) have shown early promise as potential therapeutics capable of eliciting endogenous antibodies that circulate in human serum to target TAAs.^78–80^ ARMs are bispecific molecules containing both an antibody-binding ligand and a target-binding ligand, thus redirecting an associated antibody to diseased cells or tissues.^79^ Recruited antibodies subsequently elicit an immune response through multiple pathways, including antibody-dependent cellular cytotoxicity and phagocytosis or antibody-dependent complement lysis.^81,82^ While this is a nascent field, there is one ARM currently in early phase clinical trials capable of recruiting NK cells to CD38-overexpressed multiple myeloma cells.^83^

In preclinical work, ARMs have been designed with diverse immune response-eliciting ligands such as antibody-binding peptides, aptamers,^84,85^ and small molecule haptens, such as 2,4-dinitrophenyl (DNP),^86–88^ fluorescein,^89^ and an α-1,3-galactose oligosaccharide.^90^ These motifs have been tethered to small molecules, peptides, and aptamers targeting TAAs, including folate receptor α,^84,89^ HER2,^85^ integrins,^91^ PSMA,^88^ and others.^79^ While most ARMs tether the antibody-binding domain to the antigen-targeting domain through flexible linkers, covalent TAA modification has been reported as well.^87^ Here, marking of the target antigen recruits endogenous anti-DNP antibodies to diseased cells to initiate a selective cytotoxic response.^86^ We hypothesized that functionalizing our covalent aptamers with a transferrable DNP would allow for selective marking of PTK7- or cMet-expressing cells for targeted lysis.^86^ The DNP motif was chosen as it is synthetically very accessible, in contrast to other epitopes such as glycans or peptide mimetics, and since the corresponding antibodies are abundant in human serum.^78^

The DNP LDNASA electrophile **3** was assembled in three steps from the same LDNASA core that was used for the biotin-transferring electrophile **2** and conjugated to alkyne-bearing aptamers as previously described (Figure 5A, Supporting Information Figure S11).^40^ After hapten transfer, complement system activation via component C1 recognition of the Fc domain of IgGs generates a cascade of cleavages, eventually coating the cell surface in complement products, stimulating phagocytosis and cytotoxic killing by innate immune cells.^92^

**Figure 5:**
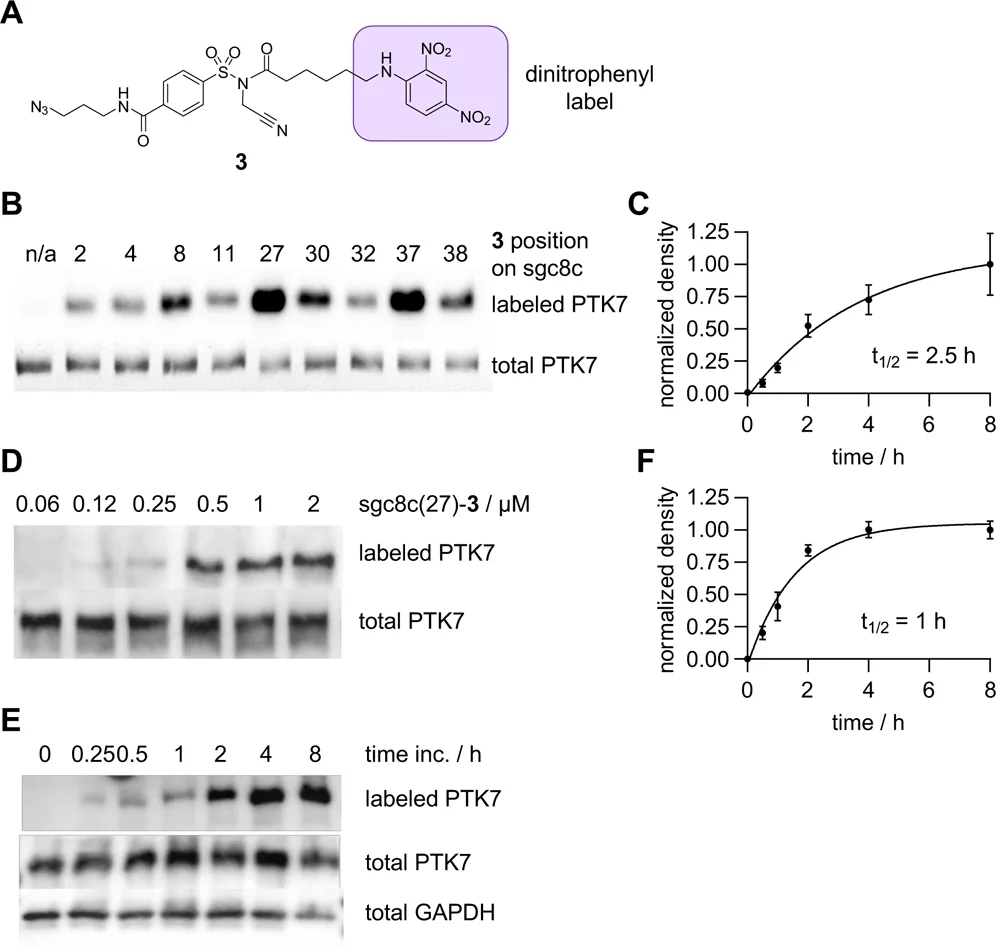
(A) Structure of the 2,4-dinitrophenyl (DNP) delivering warhead **3**. (B) SAR analysis of **3** positioning on sgc8c (0.25 μM) to label recombinant PTK7 (0.1 μM). (C) Quantification of *t*_1/2_ of DNP transfer to recombinant PTK7 (0.1 μM) using sgc8c(27)-**3** (0.25 μM). (D) Dose response of DNP transfer to HEK/PTK7 cells incubated with increasing concentrations of sgc8c(27)-**3** for 2 h. (E) Representative blot and (F) quantification of *t*_1/2_ determination of DNP transfer to HEK/PTK7 cells by sgc8c(27)-**3** (0.5 μM). DNP transfer was visualized via anti-DNP blot. Total protein was visualized via silver stain (recombinant) or anti-GFP blot (cell surface). Data represents average and error bars represent standard deviation from either two or three independent experiments.

First, we verified that changing the chemical makeup of the label appended to the cleavable electrophile did not affect the affinity of the aptamers for its target protein. An SAR study of sgc8c-mediated DNP transfer to recombinant PTK7 was performed under conditions matching the previous SAR of biotin transfer (Figure 5B).^41^ Position 27 remained the most efficient as determined by an anti-DNP blot, with positions 8, 30, and 37 additionally displaying good efficiency.^41^ Kinetic analysis of DNP transfer to recombinant PTK7 (0.1 μM) by sgc8c(27)-**3** showed similar quick transfer to that of biotin (*t*_1/2_ of labeling of 2.5 h, compared to *t*_1/2_ of ∼2 h for biotin transfer, Figure 5C, Supporting Information Figure S12).^41^

Transfer of DNP to HEK/PTK7 cells was attempted via titration of sgc8c(27)-**3** for a 2 h incubation. Cell lysates were evaluated for DNP modification by an anti-DNP blot, in which a plateau of DNP transfer to PTK7 above a 0.5 μM concentration was observed, with target specificity maintained up to a 1 μM of sgc8c(27)-**3** (Figure 5D, Supporting Information Figure S13). Using this optimized 0.5 μM concentration, a time-dependent evaluation of DNP transfer to HEK/PTK7 cells was conducted, showing apparent complete transfer after a 4 h incubation, with a calculated *t*_1/2_ of labeling of 1 h (Figure 5E,F, Supporting Information Figure S14). Here, labeling was faster with increased concentration of the covalent aptamer, similar to the observed results with biotinylation of cMet (Figure 3B).

Next, the warhead **3** was tethered to the modified cMet-targeting aptamer SL1. Because the transferred label did not impact the SAR of the PTK7-targeting aptamer, DNP transfer was attempted with the most efficient biotinylating SL1, position 29. To ensure that the identity of the label (DNP vs biotin) did not affect the affinity of SL1 for cMet, increasing concentrations of SL1(29)-**3** were added to a mixture of cMet and BSA (0.1 μM each). Specific labeling of cMet was observed with an aptamer concentration up to 1 μM (Figure S15A). Consistent with our previous publications,^40,41^ off-target labeling was observed when incubating protein with a 1 μM concentration of aptamer or higher. As this trend has been reproducible across multiple aptamer-protein pairs, it can be assumed that is the upper threshold for selective label delivery to the target protein is around 500 nM for the LDNASA warhead. However, incubation of neither sgc8c(27)-**3** nor SL1(29)-**3** with BSA at concentrations comparable to physiological serum albumin levels (∼600 μM),^93^ resulted in detectable DNP modification, supporting potential translational applicability (Supporting Information Figure S16). Kinetic analysis of DNP modification of recombinant cMet (0.1 μM) was performed by incubating SL1(29)-**3** (0.5 μM) for increasing amounts of time. Quantification showed a *t*_1/2_ of 2 h, which was longer than biotin transfer at the same concentration (Supporting Information Figure S15B,C). However, because efficient transfer was still detected, the aptamer was advanced to live cell studies.

Selective transfer of DNP was observed to HEK/cMet cells (Supporting Information Figure S15D). The *t*_1/2_ of DNP label transfer to the cell surface (2 h) did match that of recombinant cMet (2 h), indicating reproducible kinetic properties (Figure 6A). Furthermore, the successful, selective transfer of DNP to both cell-surface PTK7 and cMet showcased the versatility of covalent aptamers to modify proteins with any label compatible with the NASA synthesis. Additionally, the identity of the label (biotin vs DNP) did not impact the efficiency of transfer, supporting the notion that transfer is governed by the protein-aptamer interaction.

**Figure 6:**
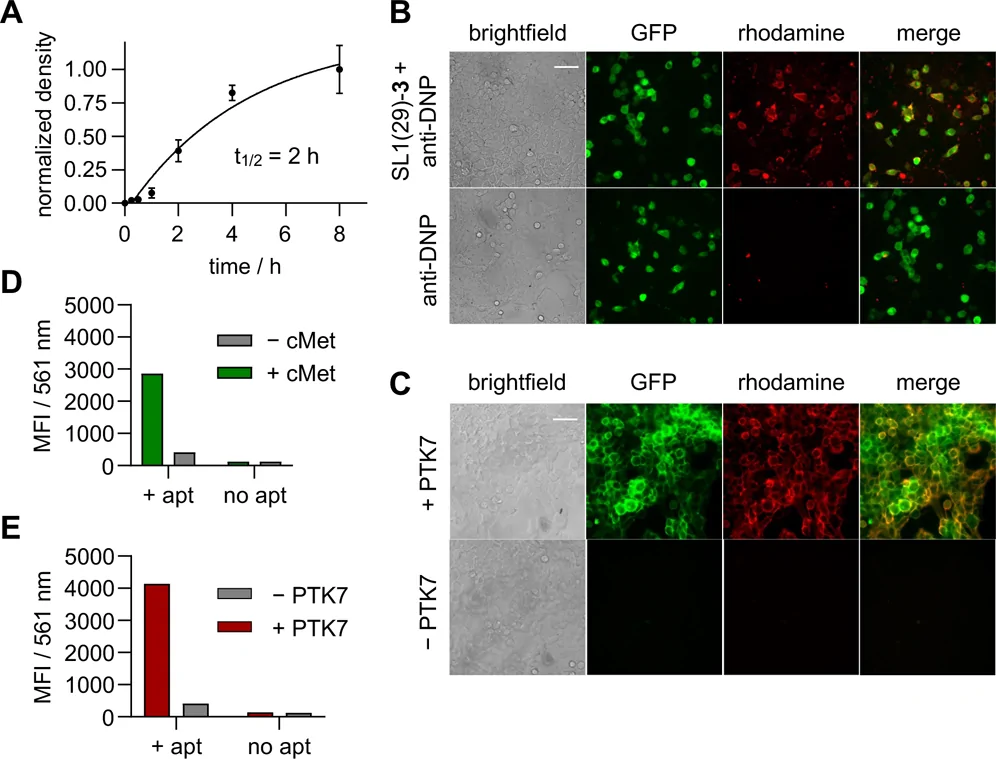
(A) Quantification of t1/2 determination of DNP transfer to cMet-GFP expressing cells. Data points represent averages and error bars represent standard deviation of triplicate data. Mean fluorescence intensity of flow cytometry analysis of (B) cMet-GFP and (C) PTK7-GFP HEK cells labeled with 0.5 μM of the appropriate DNP-transferring aptamer 2 h and subsequently incubated with 10 μg/mL TAMRA-anti-DNP for 20 min on ice. For flow cytometry, 30,000 events were recorded. Fluorescence imaging analysis of (D) cMet-GFP and (E) PTK7-GFP cells labeled in the same manner. Scale bar = 50 μm.

With successful DNP transfer detected by western blot, the ability for the installed DNP modification to recruit an anti-DNP antibody to the cell surface was evaluated. An anti-DNP antibody was stochastically labeled with NHS-TAMRA and fluorophore modification was confirmed via in-gel fluorescence (Supporting Information Figure S17). cMet expressing HEK cells were incubated with SL1(29)-3 (0.5 μM) for 2 h to install the DNP modification, excess aptamer was washed off, and then cells were incubated with 10 μg/mL TAMRA-labeled anti-DNP antibody and imaged (Figure 6B). For HEK/cMet cells, cMet (GFP) and anti-DNP (TAMRA) were found to be colocalized on cell membranes, while cMet-negative cells displayed no red fluorescence. Therefore, DNP transfer with the covalent SL1 and subsequent antibody recruitment to HEK/cMet cells was selective (Supporting Information Figure S18A) and resulted in a 7-fold increase in antibody binding to HEK/cMet cells compared to cMet-negative cells in the same population, with 86% of cMet-positive cells showing successful DNP modification (Figure 6D, Supporting Information Figure S19A,B). The same antibody was recruited to HEK/PTK7 cells labeled with sgc8c(27)-**3** (0.5 μM, 2 h incubation), resulting in colocalization of PTK7 (GFP) and anti-DNP (TAMRA) comparable to that of the cMet-expressing cells (Figure 6C, Supporting Information Figure S18B) and a 30-fold increase in antibody binding to HEK/PTK7 cells over HEK cells, with 93% of HEK/PTK7 cells showing DNP modification (Figure 6E, Supporting Information Figure S19C,D). The same quantitative colocalization analysis as was performed for biotin transfer was performed for the DNP and receptor signals, resulting in Pearson’s coefficients of 0.85 (cMet) and 0.93 (PTK7), again indicating strong colocalization.

Successful DNP modification of both biomarkers led us to test complement-dependent toxicity of the TAA-expressing cells, a major mechanism for activation of an innate immune response. Complement activation was assessed by incubating sgc8c(27)-**3** or SL1(29)-**3** with HEK cells expressing the appropriate TAA and wild-type HEK cells for 2 h. Remaining aptamer was removed and cell medium containing complement from baby rabbit serum spiked with 50 μg/mL of either anti-DNP or an off-target anti-HPV antibody (targeting the E6 protein) was added and incubation was continued for 6 h at 37 °C, followed by a an XTT cell viability assay (Figure 7A).^94^ A significant decrease in viability was observed for DNP-modified HEK/PTK7 cells treated with the anti-DNP antibody (Figure 7B). There was no significant effect on viability resultant from antibody or aptamer treatment alone or with a combination of aptamer and off-target antibody. The lack of cytotoxicity observed with off-target antibody treatment supported that binding of the target epitope was crucial for activation of the complement system. A comparable decrease in viability was observed upon anti-DNP and complement treatment of DNP-modified HEK/cMet cells (Figure 7C, Supporting Information Figure S20), determined via loss of GFP expression as previously described. Similarly, no significant toxicity was observed in any of the control treatments, indicating the necessity for the ternary complex to form in order for lysis to occur. Specifically, no significant cytotoxicity was observed from aptamer incubation alone. In addition, DNP itself is known to be nontoxic unless proper display on cell surfaces elicits an immune response.^95,96^ Therefore, targeted cell killing was achieved in two distinct cell populations without the need for T cell engineering.

**Figure 7:**
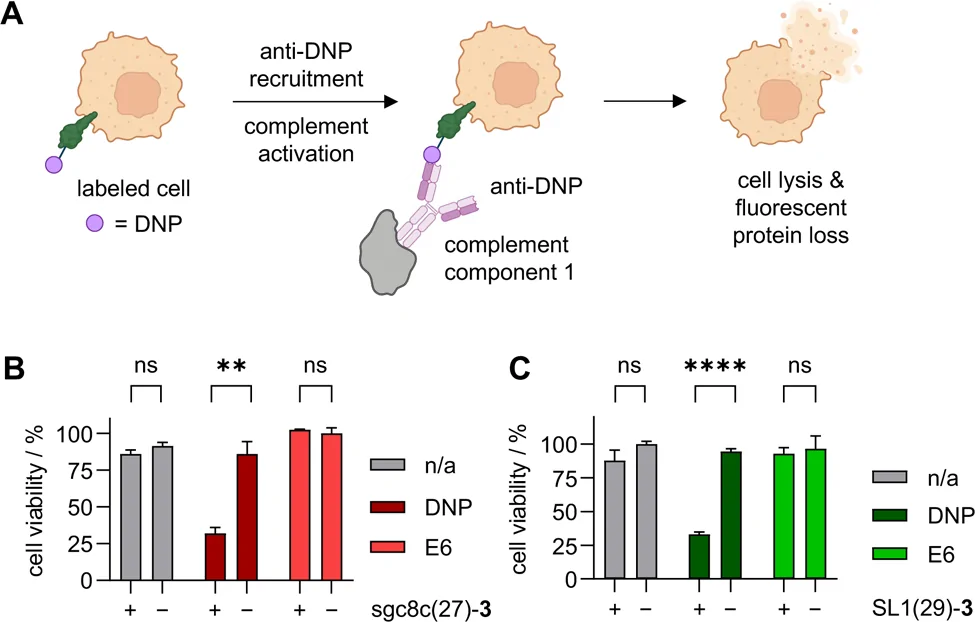
(A) Schematic depicting complement-dependent cytotoxicity of anti-DNP labeled cells. (B) Selective lysis of HEK/PTK7 and (C) HEK/cMet cells labeled with the appropriate DNP-transferring aptamer (0.5 μM, 2 h) and subsequently incubated with 10% [v/v] baby rabbit complement and 50 μg/mL of the indicated antibody for 6 h. Cell viability of HEK/PTK7 cells was read out via an XTT assay. Cell viability of HEK/cMet cells was determined as a factor of the percentage of GFP-expressing cells in the entire population. Bars represent averages and error represents standard deviation of three independent experiments. ns = not significant. ** = *p* < 0.005. **** = *p* < 0.0001.

## Summary and Conclusion

Aptamers have gained momentum and shown promise as therapeutics and diagnostic agents but have not found widespread clinical success, possibly due to their short target engagement times, propensity toward nuclease-mediated degradation, fast renal clearance, and short half-lives in vivo.^37,97,98^ Covalent aptamers are addressing these issues by permanently engaging the functional moiety, here a biotin or DNP motif, with a target protein. This covalent labeling extends the lifetime of functionality from the lifetime of the aptamer to the lifetime of the protein.

The development of immuno-oncology therapeutics takes advantage of endogenous and engineered immune mechanisms, redirecting a biological response toward specific antigens overexpressed on tumor cells. Numerous therapies have gained FDA-approval showing increased survival compared to traditional chemotherapeutic techniques. Here, covalent aptamers were developed to directly tag cells with an adaptor molecule to mark TAA-expressing cells to stimulate an immunologic response. Utilizing our previously established PTK7-targeting covalent aptamer, HEK/PTK7 cells were biotinylated and then targeted by universal mSA2-CAR T cells, leading to specific lysis. Additionally, PTK7 cells were marked with a DNP molecule, allowing for recruitment of anti-DNP antibodies known to endogenously circulate. Binding of the anti-DNP antibody triggered complement system activation and antibody-dependent complement cytotoxicity, again leading to selective death of HEK/PTK7 cells. The covalent aptamer technology was expanded to target cMet through functionalization of its established targeting aptamer, SL1,^47^ with selectivity that rivaled that of PTK7. This new covalent aptamer was able to mark HEK/cMet cells for immune response utilizing the same biotin- and DNP-transferring electrophiles as sgc8c, showing similar activation and lysis levels. This target expansion demonstrates the generalizability of our covalent aptamer methodology. PTK7 and cMet are both prevalent cancer biomarkers in which overexpression and/or dysregulation is correlated with poor prognosis, making them excellent candidates for immunotherapy technology. To our knowledge, this is the first instance in which the target cell was directly modified with the binding epitope of a universal CAR utilizing a nucleic acid ligand.

While this platform is in principle expandable to any protein for which an aptamer ligand exists, factors such as the number of accessible lysine residues in proximity to the aptamer-binding epitope may influence labeling efficiency. Proteins in which reactive residues are sterically occluded or located distal to the aptamer-binding interface may therefore be less amenable to efficient labeling.^67^ In addition, cell-surface receptors represent particularly favorable targets, whereas targeting intracellular proteins, while possible, would require additional strategies and optimization for aptamer delivery to the appropriate cellular compartment.^99^ In addition, chemical modification of the nucleic acid scaffold to improve nuclease resistance and pharmacodynamic properties represents an important future direction.^37^ Because aptamers can be readily synthesized with diverse chemical modifications, this platform should be broadly compatible with unnatural nucleic acids. The assembly of our electrophile-modified aptamers is easier, cheaper, and more reproducible than site-selective antibody modification due to the ease of solid phase oligonucleotide synthesis.^100–102^ Additionally, aptamer treatment does not produce any of the potential immunogenic effects caused by antibody treatment, altogether introducing a less expensive and possibly safer method for universal CAR T cell treatment.^27,98^

Our approach was expanded to showcase the covalent transfer of a new label, DNP, capable of recruitment of an endogenous antibody.^86,87^ Engagement of the corresponding antibody activated complement system proteins and led to a cytotoxic response, resulting in selective death of cancer antigen-expressing cells.^81^ Taken together, two different methods of immune activation and selective cell killing through aptamer targeting were achieved. This approach can be expanded to any protein with an aptamer partner. Additionally, the consistency observed between biotin transfer and DNP transfer validated that the kinetics and efficiency of labeling are dependent on the aptamer and not on the identity of the label.

## Supplementary Material



## Notes

Experimental details and methods; Supporting Information Figures; plasmid maps; lists of primer and aptamer sequences; and ^1^H and ^13^C NMR spectra (PDF)
