# Supplementary material for: Targeting Adaptive and Innate Immune Responses with Covalent Aptamers
Source: J Am Chem Soc. 2026 Aug 10;148(32):34928–37. doi: 10.1021/jacs.6c10475 (PMC13495756; doi:10.1021/jacs.6c10475)
Supplement: Supplementary file 1 [file ja6c10475_si_001.pdf]

Supporting Information

Targeting Adaptive and Innate Immune Responses with Covalent Aptamers

Savannah Albright[a], Avani Parikh[b] Mary Cacace[a], Carolyn Rosenblum[a], Michael Dorogan[a], Jason Lohmueller[b,c], and Alexander Deiters*[a,c]

[a] Department of Chemistry, University of Pittsburgh, Pittsburgh, PA 15260, United States
[b] Department of Surgery, Division of Surgical Oncology, University of Pittsburgh, Pittsburgh, PA 15213, United States
[c] Institute for Synthetic Biology, University of Pittsburgh, Pittsburgh, PA 15260, United States

Supporting Methods

Chemical Synthesis

See **Figure S11** below for the synthetic scheme.

***N*-(3-Azidopropyl)-4-(*N*-(6-((2,4-dinitrophenyl)amino)hexanoyl)sulfamoyl)benzamide.** *N*-(3-azidopropyl)-4-sulfamoylbenzamide (100 mg, 0.37 mmol) synthesized as previously described,[1] 6-((2,4-dinitrophenyl)amino)hexanoic acid (164 mg, 0.55 mmol), EDC (213 mg, 1.11 mmol), TEA (155 µL, 1.11 mmol), and DMAP (9 mg, 0.07 mmol), were dissolved in DMF (5 mL). The reaction mixture left stirring at room temperature for 16 hours then diluted with DCM (20 mL) and was washed with brine (50 mL, 3X). The organic layer was then dried over anhydrous sodium sulfate and concentrated. The crude mixture was purified by silica gel chromatography (50-100% EtOAc/Hex). The product was isolated as a yellow solid (56%, 117 mg). ^{1}H (300 MHz, DMSO) δ 1.27-1.19 (m, 2H), 1.57-1.40 (m, 4H), 1.78 (quin, 2H, J = 6.78), 2.23 (t, 2H, J = 7.26), 3.34-3.30 (m, 2H), 3.43-3.36 (m, 4H), 7.17 (d, 1H, J = 9.69), 8.03-7.95 (m, 4H), 8.24 (dd, 1H, J = 2.66, 6.91), 8.85-8.72 (m, 3H), 12.16 (s, 1H). ^{13}C (500 MHz, DMSO) δ 23.50, 25.43, 27.62, 28.21, 35.17, 36.76, 42.50, 48.46, 115.19, 123.68, 127.49, 127.89, 129.59, 129.92, 134.61, 139.00, 141.35, 148.06, 165.10, 171.61. HRMS (ESI+) calcd for $C_{22}H_{25}N_8O_8S$ $(M-H)^-$ 561.15106, found: 561.15169.

***N*-(3-Azidopropyl)-4-(*N*-(cyanomethyl)-N-(6-((2,4-dinitrophenyl)amino)hexanoyl)sulfamoyl) benzamide (3).** TEA (30 µL, 0.20 mmol) and iodoacetonitrile (30 µL, 0.40 mmol) were added to a solution of the benzamide (20 mg, 0.04 mmol) in dry DMF (500 µL). The reaction mixture was stirred at room temperature for 24h in the dark. The residue was concentrated and purified by silica gel chromatography (0-5% MeOH/DCM). The product was isolated as a yellow solid (47%, 11 mg). ^{1}H (500 MHz, DMSO) δ 1.32-1.26 (m, 2H), 1.59-1.48 (m, 4H), 1.78 (quin, 2H, J = 6.78), 2.67 (t, 2H, J = 7.17), 3.33 (q, 2H, J = 6.45), 3.44-3.40 (m, 3H), 5.00 (s, 2H), 7.19 (d, 1H, J =9.68), 8.07 (d, 2H, J = 8.60), 8.12 (d, 2H, J =8.62), 8.24 (dd, 1H, J = 9.61, 2.69), 8.803 (q, 2H, J = 6.35), 8.85 (d, 1H, J = 2.73). 13C (500 MHz, DMSO) δ 23.40,25.29, 27.65, 28.17, 33.94, 34.95, 36.82, 42.51, 48.46, 115.19, 116.33, 123.67, 127.82, 128.37, 129.60, 129.92, 134.62, 139.79, 139.92, 148.0,7 164.71, 171.96. HRMS (ESI+) calcd for $C_{24}H_{27}N_9O_8S$ $(M+H)^+$ 602.17761 found: 602.17557.

Oligonucleotide Synthesis. Prior to May 2023, oligonucleotide syntheses were performed as previously described using standard β-cyanoethyl phosphoramidite chemistry on an Expedite 8909 DNA/RNA Synthesizer (Distribio, NY, USA).[2] Briefly, aptamers were synthesized on a 200 nanomole scale using 500 Å derivatized CPG solid phase supports obtained from Glen Research (VA, USA) using a 5'-dimethoxytrityl (DMTr)-OFF approach. Synthesis cycles with coupling times of 2 and 10 minutes were used for both unmodified and modified phosphoramidites (**1**) respectively, at 0.07 M concentration. Coupling efficiency was monitored by DMTr cation release after each deprotection step – no significant loss was observed following modified phosphoramidite addition to the oligonucleotide.

After May 2023, oligonucleotides were purchased from either the core research facilities at the University of Utah or IDT and received as lyophilized solids. Purchased oligonucleotides were resuspended in nuclease-free milliQ water at a concentration between 50 - 100 μM and stored at −20 °C as 50 μL aliquots.

Reagent and phosphoramidite list (Glen Research, VA, USA). dA-CE phosphoramidite (10-1000), Ac-dC-CE phosphoramidite (10-1015), dmf-dG-CE phosphoramidite (10-1029), dT-CE phosphoramidite (10-1030), tetrahydrofuran/2,6-lutidine/acetic anhydride (40-4010), 5-ethylthio-1H-tetrazole (ETT, 30-3040), 0.02 M iodine in tetrahydrofuran/pyridine/water (88:10:2)(40-4032), 3% dichloroacetic acid in dichloromethane (40-4040), and anhydrous acetonitrile (40-4050).

Oligonucleotide deprotection. Cleavage of the oligonucleotide from the solid support and subsequent deprotection were performed as previously described.[2] Briefly, deprotection was carried out with 1 mL of 2 N ammonia in methanol at room temperature for 1 h. Following, concentration *in vacuo,* the clear oil was resuspended in 1:1 methylamine in ammonium hydroxide (1 mL) and heated at 65 °C for 15 min. The solution was then concentrated *in vacuo* prior to isopropanol precipitation.

Ethanol precipitation of modified oligos. Oligonucleotide precipitation was performed as previously described.[2] Briefly, precipitation was achieved through addition of sodium acetate (3 M, pH 5.2, 0.1 volume worth) and 100% ethanol (3 volumes worth) to the oligonucleotide solution (10-200 μM, 1V) in milliQ water. The solution was vortexed a d cooled at –80 °C for 30 min and subsequently pelleted (13.2 rpm, 15 min, 4 °C. The supernatant was discarded and the pellet washed with 70% ethanol (3V) and pelleted as described above. The supernatant was removed, and the pellet resuspended in milliQ water. The oligonucleotide concentrations were determined by a NanoDrop Spectrophotometer (ND1000). Oligonucleotide purity was analyzed by HPLC, using a gradient of 5-30% buffer B over 30 min at a flow rate of 1 mL/min on an ACE oligo C18 column (4.6 x 100 mm, 3 μM particles). The buffers used were 0.1 M TEAA (Buffer A) and ACN (Buffer B). The column oven was set to 40 °C.

Conjugation of the azide-tethered electrophile to the alkyne-modified oligonucleotide. Copper catalyzed [3+2] cycloaddition (click) reactions were performed as previously described. Briefly, to 50 μL of the alkyne-bearing oligo (20-200 μM in milliQ water) was added 5 μL of triethylammonium acetate buffer (2 M, pH 7, Glen Research) and DMSO (55 μL) before the solution was vortexed. Following 5 μL of the azide electrophile (10 mM in DMSO) was added and the reaction mixture was vortexed. To the resulting solution was added 12 μL of a freshly made ascorbic acid solution (5 mM in deionized water) and the reaction mixture was vortexed again. Argon gas was bubbled through the reaction mixture via a needle for thirty seconds and the tube cap was quickly closed. Lastly, 6.5 μL of copper (II)-tris[(1-bennzyl-1*H*-1,2,3-triazol-4-yl)methyl]amine (TBTA, 10 mM in 55% DMSO) was quickly added, the reaction mixture was vortexed and incubated at room temperature for 1 h. Reaction progression was monitored by

HPLC using method/conditions described previously. If the click reaction did not proceed to completion after 4 h, products were further purified by HPLC and lyophilized. The dried oligonucleotides were then resuspended in 50 μL of milliQ water and concentrations were determined by NanoDrop (ND1000).

Laemmli loading buffer recipe (4X). Tris buffer (1 M, pH 6.8, 15 mL), SDS (6 g), glycerol (30 mL), 2-mercaptoethanol (15 mL), and bromophenol blue (0.1 g) were combined in a 50 mL conical tube. Deionized water was added to 40 mL and the solution was vortexed until the solids dissolved before the remaining 10 mL were added. The buffer was aliquoted and stored at –20 °C.

Aptamer-mediated biotin transfer to recombinant protein. Recombinant PTK7 (R&D Biosystems 9799-TK) and cMet (R&D Biosystems 8614MT100) were dissolved at 4.2 μM and 1.9 μM, respectfully, in DPBS supplemented with 5 mM $MgCl_2$ and 4.5 g/L glucose, pH 7.4 (supplemented DPBS) following the manufacturer's recommendations. As an off-target protein, BSA (Thermo Fisher, AAJ1085722) was dissolved to a concentration of 10 μM in supplemented DPBS. A master mix containing 0.1 μM of the appropriate target protein and 0.1 mg/mL yeast tRNA (Fisher, 50-111-8112) as a blocking agent in supplemented DPBS was generated and scaled appropriately for the number of reactions such that the total volume for each reaction was 20 μL. For example, to generate a master mix for a cMet labeling reaction with 10 conditions, recombinant cMet (10.5 μL) and yeast tRNA (2 μL) would be diluted to 200 μL in supplemented DPBS (187.5 μL). If performing a selectivity experiment, the master mix was supplemented with the BSA solution (2 μL) in place of the corresponding volume of supplemented DPBS. The master mix was split into 18 μL in 0.2 mL PCR tubes.

To initiate labeling, a 20 μM aptamer solution was made in supplemented DPBS. For additional aptamer concentrations, the 10 μM stock was further diluted to achieve 5, 2.5, 1.25, 0.63 μM (10X) working stock solutions. Labeling was initiated by the addition of the aptamer solution (2 μL) to the PTK7 solution to a final reaction volume of 20 μL. Reactions were incubated at 37 °C for the indicated amount of time and quenched by addition of 4X Laemmli buffer (7 μL) and heating at 95 °C for 10 minutes.

Analysis of labeled proteins *in vitro.* Following aptamer-mediated biotin transfer and quenching, samples (10 μL) were loaded onto two 6% SDS-PAGE gels, and separated via electrophoresis (60 V 20 min, 150 V 1 h). Proteins were transferred (80 V, 2 h) to a PVDF membrane (GE Healthcare) and the membrane was incubated in blocking buffer (5% BSA in TBS with 0.1% [v/v] Tween 20) for 1 h at room temperature. The blots were probed with streptavidin-HRP (1:10000 dilution, Thermo Fisher N100) in 1X TBS with 0.1% (v/v) Tween 20 (TBST, 10 mL) at room temperature for 1 h while rocking. Blots were then washed three times with TBST (10 mL, 5 min) at room temperature while rocking. To develop chemiluminescence, blots were incubated with equal volumes of solution A (4.4 mM luminol and 4.3 mM 4-iodophenol in 0.1 M Tris pH 9.35) and solution B (0.1 M Tris pH 9.8 with 0.01% hydrogen peroxide) for (6 mL total, 5 min) at room temperature with rocking. The blots were imaged on a ChemiDoc Imaging System (BioRad) using automated exposure settings. To visualize the total protein loaded, gels were developed with silver stain (Pierce, ThermoFisher 24162) according to the manufacturer's protocol and imaged on ChemiDoc Imaging system using automated exposure settings.

Following aptamer-mediated DNP transfer and quenching, the same SDS-PAGE and transfer techniques were followed as for biotinylated samples. However, following the initial incubation in blocking buffer, the blots were probed with diluted anti-DNP (1:2500, ThermoFisher A150-117A) in the 5% BSA in TBST blocking buffer at 4 °C overnight while rocking. Blots were then washed three times with TBST (10 mL, 5 min) at room temperature while rocking. Blots were then probed

with anti-goat (1:5000, Proteintech, SA0000I-2) in TBST at room temperature for 1 h while rocking. Blots were then washed an additional three times with TBST (10 mL, 5 min) and chemiluminescence and total protein were visualized as for biotinylated samples.

For quantification, bands were integrated using ImageJ by drawing rectangles around lanes and analyzed using the plot lanes function. Band integrations were normalized to the loading control.

Fluorescent modification of anti-DNP. Modification of anti-DNP was performed using 5 eq. of 5(6)-TAMRA SE (Ambeed, A718529). The anti-DNP (50 μL of 1 mg/mL or 3.33 μM stock) was diluted with 1 M sodium borate pH 8.5 (10 μL), milliQ water (20 μL), and DMSO (3.33 μL). A 10 mM stock of 5(6)-TAMRA SE was diluted to 100 μM by performing 2, 10x dilutions (10 μL of 5(6)-TAMRA SE into 90 μL of DMSO). The diluted 5(6)-TAMRA SE was added to the antibody solution (16.67 μL) such that the final concentration was 16.67 μM (5 eq.) and the final DMSO concentration was 20% in the 100 μL total volume. The labeling reaction was incubated for 1 hour at room temperature.

Labeled antibody was purified using a 50 kDa molecular weight cutoff filter (MWCO, Sigma, UFC5050) by diluting the labeling reaction to 500 μL with PBS, adding to the MWCO, and centrifuging at max speed for 10 minutes. The MWCO was then inverted and the volume remaining in the spin cup was centrifuged into a clean, 1.7 mL microcentrifuge tube at max speed for 2 minutes. The collected volume was diluted to 50 μL in PBS, the concentration checked on the nanodrop. Although fluorescence could be observed by eye as the solution turned pink, in gel fluorescence was checked by SDS-PAGE (10% gel) and quantified using BSA standards. The antibody was diluted 5x (2 μL of antibody into 8 μL of PBS), denatured through addition of 4x Laemmli buffer (3.33 μL) and heating at 95 °C. Samples were electrophoresed at 60 V for 20 minutes, then 150 V for 1 hour. In gel fluorescence was imaged on the ChemiDoc using the automatic exposure setting on the “rhodamine” protocol. Total protein was visualized using coomassie stain.

Plasmid construction. Top 10 chemically competent cells were used for all cloning. The pLenti-MetGFP construct was purchased from AddGene (# 37560). See **Supporting Table S3** for a list of primers used. Maps of cloned plasmids are presented in **Supporting Figure S21**. The PTK7 coding sequence was PCR amplified from pcDNA3-PTK7-CFP[2] using primers P1 and P2 and cloned into the pLenti-MetGFP, which was amplified using primers P3 and P4. All PCR was performed using Phusion (Thermo Fisher, F534L) according to the manufacturer’s suggested protocol. PCR products were gel purified (Thermo Fisher, K0692) according to the manufacturer’s protocol. The two-fragment Gibson assembly reaction was set up using 100 ng of the pLenti-GFP amplicon and a 3:1 ratio of the PTK7 amplicon following the previously reported protocol.[3] The reaction was incubated at 50 °C for 1 hour and then treated with Dpn1 (NEB) overnight prior to transformation. The reaction was then transformed into Top10 competent cells. The construct was confirmed by Sanger Sequencing (Azenta) using the available “EGFP-N” forward and “BGHR” reverse sequencing primers.

Cell culture maintenance and transfection. All cell culture experiments were performed in a sterile laminar flow hood. HEK293T and HEK:PTK7-GFP cells were maintained in Dulbecco’s Modified Eagle Medium (DMEM) supplemented with 10% (v/v) fetal bovine serum (FBS), 1% (v/v) penicillin/streptomycin (p/s), and 0.2% plasmocin prophylatic at 37 °C with 5% CO2. Cells were used between passage number 6 and 30. Cell lines were tested for mycoplasma contamination using the MycoScope PCR Detection Kit (Genlantis, MY01100) every 4 months.

Cells expressing cMet-GFP were transiently transfected using Lipofectamine 3000 following a modified version of the manufacturer's protocol. The transfection was scaled up depending on the size and number of wells being transfected. Pipetting volumes are included in **Supporting Table S1**. Once cells reached 80-90% confluency, transfection reagents were prepared by diluting plasmid with P3000 into Opti-Mem transfection media (OMEM) in a 1.7 mL microcentrifuge tube. In a separate 1.7 mL microcentrifuge tube, Lipofectamine 3000 was diluted into OMEM. The separate reagents were incubated at room temperature for 5 min. Then, the transfection reagents were combined into a single 1.7 mL microcentrifuge tube, pipetted up and down one time to mix, and incubated at room temperature for an additional 20 min. The appropriate volume of transfection reagent was added to appropriate wells. Cells were incubated for 24 hours at 37 °C with 5% CO_2 before being used for the next step.

Stable cell line generation. HEK:PTK7-GFP and HEK:cMet-GFP stable cell lines were generated by transducing 293 HEK-T cells with cMet-GFP (AddGene #37560) or PTK7-GFP (cloned) lentivirus, respectively, using polybrene. Transduced cells underwent fluorescence-activated cell sorting (FACS) for GFP expression. Post FACS, cells were expanded and checked for GFP expression before using them for experiments. Cells were maintained in DMEM + 10% FBS + 1% penicillin-streptomycin + 1X MEM amino acid solution, 10mM sodium pyruvate.

Primary human T cell culture and gamma-retrovirus transduction. All primary T cells for experiments were sourced from deidentified human Buffy Coat samples purchased from the Pittsburgh Central Blood Bank fulfilling the basic exempt criteria 45 CFR 46.101(b)(4) in accordance with the University of Pittsburgh IRB guidelines. PBMC were isolated from Buffy Coats from healthy volunteer donors using Ficoll gradient centrifugation, and human T cells were isolated using the Human Pan T cell isolation kit (Miltenyi Biotec). Human T cells were cultured in supplemented RPMI media; however, 10% Human AB serum (Gemini Bio-Products) was used instead of FBS, and the media was further supplemented with 100 U/ml human IL-2 IS (Miltenyi Biotec), 1 ng/ml IL-15 (Miltenyi Biotec), and 4 mM L-arginine (Sigma Aldrich). T cells were stimulated and expanded using TransAct Human T cell activation reagent (Miltenyi Biotec). For transduction, 48 h after activation, isolated T cells were transduced by spinning cells on retronectin (RN) (Takara Bio) coated plates. To coat the plates, 10 µg/mL RN in PBS was added to a non-tissue culture coated plate at 4 °C for 24 h. After 24 h, RN was removed, and 2-4 mL of viral supernatant was added to each well and spun at 2000 x g for 2 h at 32 °C. Post 2 h of spinfection, 2 mL of media was removed, and 1x 10^6 T cells in 4 mL were added per well and spun at 32 °C for 10mins at 1000 g. 24 h after spinfection, each well received 100 U/mL IL-2 and 1 ng/mL IL-15. After 72 h, cells were moved to a 6-well tissue culture coated plate and maintained at a concentration of 1x 10^6 cells and supplemented with fresh 100 U/mL IL-2 and 1 ng/mL IL-15. Cells were split to a concentration of 10^6/ mL and supplemented with fresh IL-2 and IL-15 every 2–3 days. After 10-12 days of stimulation and expansion, transduced cells were evaluated for CAR expression by flow cytometry and evaluated for activity in subsequent functional assays.

Protein labeling and western blot analysis. Cells (50,000 per well) were seeded into a 48-well plate in 250 µL of DMEM + 10% FBS. If transfection was necessary, when cells reached 80-90% confluency (typically 24 hours), cells were transfected according to **Supporting Table S1** and incubated for another 24 hours. After 48 hours post-plating, cells were washed three times with 200 µL of room temperature supplement DPBS. Labeling was performed by incubating with 100 µL of the indicated concentration of aptamer diluted in supplemented DPBS containing 0.1 mg/mL yeast tRNA for the indicated time length at 37 °C. Cells were then washed three more times with 100 µL of supplemented DPBS, lysed with 65 µL RIPA buffer (Fisher) containing 100x HALT protease inhibitor (ThermoFisher), and stored at –80 °C until further use.

SDS-PAGE and western blot analysis of labeled proteins. Protein samples (60 µL) were boiled in 4X Laemmli buffer (20 µL) for 10 minutes at 95 °C. SDS-PAGE gels (6%/10% (v/v)) were loaded with 10 µL each sample, which were separated via electrophoresis (60 V, 20 min then 150 V, 1 h). Afterwards, proteins were transferred (80 V, 2 h) to a PVDF membrane (GE Healthcare) and the membrane was blocked in blocking buffer (5% in 1X TBS with 0.1% [v/v] Tween 20 [TBST]) for 1 h. Blots were probed for biotinylation with streptavidin-HRP or anti-DNP dependent on the label transferred accordingly to the previously described protocol for recombinant protein. Anti-GFP and anti-GAPDH control blots were incubated with anti-GFP rabbit polyclonal (Proteintech, 50430-2-AP, 1:5000 dilution in TBST) and anti-GAPDH rabbit polyclonal (Proteintech, 10494-1-AP, 1:5000 dilution in TBST) antibodies, respectively, at 4 °C overnight with rocking. Blots were washed three times with TBST (5 mL, 5 min) at room temperature with rocking before incubating with anti-rabbit (Proteintech, SA00001-2, 1:5000 dilution in TBST) for at room temperature for 1 h with rocking. Chemiluminescence was visualized and quantified as previously described. Band integrations were normalized to the loading control.

Live cell fluorescence imaging. Cells (25,000 per well) were seeded into a 96-well imaging plate in 100 µL of DMEM + 10% FBS and incubated overnight at 37 °C with 5% CO_2. If transfection was necessary, when cells reached 80-90% confluency (typically 24 hours), cells were transfected according to **Supporting Table S1** and incubated for another 24 hours. After 48 hours post-plating, cells were washed three times with 100 µL of wash buffer. Labeling was performed by incubating in 50 µL of the appropriate aptamer solution at the indicated concentration in binding buffer for 2 h, unless otherwise specified, at 37 °C with 5% CO_2. Cells were washed three more times with 100 µL of wash buffer. Biotinylated cells were then incubated with 50 µL of 10 µg/mL neutravidin-tetramethylrhodamine (NA-TAMRA, Fisher, A6373A) in binding buffer for 5 minutes at room temperature. DNP-labeled cells were incubated with 50 µL of 10 µg/mL TAMRA-labeled anti-DNP in binding buffer for 20 minutes on ice. Cells were washed three more times with 100 µL of wash buffer. Live Cell Imaging Solution (100 µL, Invitrogen) was added and cells were imaged using a Zeiss Axio Observer Z1 with an Andor Zyla 4.2 camera, Plan-Apochromat 63x/1.4 objective, and the FITC (Chroma filter 49002, ex. ET 470/40, em. ET 525/50) and TRITC (Chroma filter 49004, ex. ET 545/25, em. ET 605/70) filter sets. Images were exported in TIFF format using SlideBook. Image processing was conducted using ImageJ. Colocalization was quantified by calculating the Pearson's correlation coefficient using FIJI (coloc2 plugin).

Flow cytometry analysis. Cells (50,000) were seeded into a 48-well plate in 250 µL of DMEM + 10% FBS and incubated at 37 °C with 5% CO_2. If transfection was necessary, when cells reached 80-90% confluency (typically 24 hours), cells were transfected according to **Supporting Table S1** and incubated for another 24 hours. After 48 hours post-plating, cells were washed and labeled with the appropriate aptamer as previously described. Cells were washed again, and the appropriate fluorescent cargo was recruited as described for fluorescence imaging but utilizing a 100 µL volume. After final washing, cells were incubated in 100 µL trypsin for 5 minutes at 37 °C with 5% CO_2 to lift from the plate, then diluted to 1 mL with DMEM + 10 % FBS Cells were then pelleted via centrifugation (500 g, 5 min) and resuspended in 200 µL supplemented DPBS. All flow cytometry experiments were run on the LSRFortessa flow cytometer (BD Biosciences) and data were collected using the BD FacsDiva software v9.0 (BD Biosciences). Flow cytometry data was analyzed using FlowJo v10.10.0 (FlowJo, LLC), and data was presented and analyzed using Graphpad Prism v10.5.0 (GraphPad Software, LLC).

CAR T cell and target cell aptamer-mediated activation co-incubation assay. HEK:PTK7-GFP and/or HEK293T cells (1,000,000 cells) were seeded in a 6-well plate in 2 mL of DMEM + 10% FBS and incubated at 37 °C with 5% CO_2. If transfection was necessary, when cells reached 80-90% confluency (typically 24 hours), cells were transfected according to **Supporting Table S1**

and incubated for another 24 hours. After 48 hours post-plating, cells were washed three times with 1 mL of supplemented DPBS. Prior to mSA2 CAR T cell addition, cells were labeled with 0.5 µM of the appropriate biotinylating aptamer for 2 hours in supplemented DPBS containing 0.1 mg/mL tRNA at 37 °C with 5% CO_2. After 2 hours, cells were washed twice with supplemented DPBS and trypsinized with 200 µL of Tryple Express (Gibco, 12604021) for 3 minutes. Trypsin activity was quenched by addition of 1.8 mL of fresh DMEM supplemented with 10% (v/v) FBS.

Labeled cells were counted and stained with Cell Trace Yellow following manufacturer's recommendation (ThermoFisher, C34567), re-suspended in RPMI, counted, and plated at 20,000 cells per well in 50 µl in a 96 well V-bottom plate. 50 µL of CAR T cells were added at the indicated E:T ratio (for example, for a 5:1 ratio, 100,000 effector cells were added). The final volume was made to 200 µL by adding RPMI media. Plates were incubated at 37 °C with 5% CO_2 for 48 hours. After incubation, cells were stained with antibodies against T cell activation marker CD69-PE (BD Biosciences) and CAR expression marker anti-CD271 (LNGFR) -BV421 (BD Biosciences) diluted at a 1:100 ratio in flow cytometry buffer (PBS + 2% FBS + 1% Penicillin-streptomycin), for 30 minutes on ice in the dark. After 30 minutes, cells were washed twice with flow cytometry buffer and evaluated for marker expression by flow cytometry. Live cells were quantified as described in target cell lysis via immune cell assay. CAR+ cells were gated on LNGFR expression, and CD69 mean fluorescence intensity (MFI) was plotted on LNGFR+ cells. 50,000 total events were recorded per sample. Assays were performed in triplicate. Significance was determined via Welch's unpaired t-test using Graphpad Prism v10.5.0 (GraphPad Software, LLC).

Cell culture supernatants were collected after 48 h of co-incubation, and IFN-γ levels were quantified by ELISA (Biolegend) according to the manufacturer's protocol. Recombinant IFN-γ standards were included for quantification.

Target cell lysis via immune cell assay. To identify lysed HEK: PTK7-GFP cells, samples were stained with Ghost Dye Red Viability Dye (Cytek Bio, 13-0871-T100) diluted at 1:1000 in PBS and incubated for 15 minutes at 4 °C. Target cells were identified by Cell Trace Yellow and lysed target cells were identified by positive Ghost Dye staining on Y axis and Cell trace yellow staining on X axis. Specific cytotoxicity was calculated by the equation: 100 * (% experimental lysis – % target-only lysis) / (100 – % target-only lysis). For experiments with cMet-GFP target cells, GFP was used as the marker for live transduced(cMet+) target cells in determining lysisas adapted from previously described protocols.[4] Assays were performed in triplicate. Significance was determined via Welch's unpaired t-test using Graphpad Prism v10.5.0 (GraphPad Software, LLC).

Aptamer-mediated complement activation and lysis of HEK:PTK7-GFP cells. HEK:PTK7-GFP and HEK293T cells (25,000 cells) were both seeded in a 96-well plate and incubated at 37 °C with 5% CO_2 until they reached 80-90% confluency. Cells were washed three times with 100 µL supplemented DPBS and labeled with 0.5 µM of sgc8c(27)-**3** for 2 hours in supplement DPBS containing 0.1 mg/mL tRNA at 37 °C with 5% CO_2. After 2 hours, cells were washed three times with 100 µL supplemented DPBS and incubated with 100 µL of DMEM + 10% FBS containing 10% (v/v) baby rabbit complement (Cedarlane Labs, CL3442-R) and 50 µg/mL of the appropriate antibody. Cells were incubated for 4 hours, after which viability was read out by an XTT assay. Assays were performed in triplicate. Significance was determined via Welch's unpaired t-test using Graphpad Prism v10.5.0 (GraphPad Software, LLC).

Aptamer-mediated complement activation and lysis of cMet-GFP expressing cells. HEK293T cells were seeded in a 6-well plate and incubated at 37 °C with 5% CO_2 until they reached 80-90% confluency. Cells were transfected according to **Supporting Table S1** and incubated for another 24 hours. After 48 hours post-plating, cells were washed three times with 1

mL supplemented DPBS. Prior to antibody and complement serum addition, cells were labeled with 0.5 µM of SL1(29)-**3** for 2 hours in supplemented DPBS containing 0.1 mg/mL tRNA at 37 °C with 5% CO_2. After 2 hours, cells were washed three times with 1 mL supplemented DPBS, trypsinized with 200 µL of Tryple Express (Gibco) for 3 minutes to lift from the plate and then quenched by addition of 1.8 mL of fresh DMEM + 10% FBS. Cells were counted and stained with Cell Trace Yellow following manufacturer's recommendation (ThermoFisher), re-suspended in DMEM-media and plated at 20,000 cells per well in 50 µL in a 96 well V-bottom plate. 50 µL of DMEM containing 20% (v/v) baby rabbit complement (Cedarlane Labs) and 100 µg/mL of the appropriate antibody was added to cells such that the final concentration in each well was 10% (v/v) baby rabbit complement and 50 µg/mL of the appropriate antibody. Plates underwent a quick spin to collect cells at the bottom of the wells and were incubated at 37 °C for 4 hours. To identify lysed cells, samples were stained with Ghost Dye Red Viability Dye (Tonbo Biosciences) as described earlier and analyzed by flow cytometry. Target cell viability was determined using the number of GFP-expressing cells as a viability metric. Assays were performed in triplicate. Significance was determined via Welch's unpaired t-test using Graphpad Prism v10.5.0 (GraphPad Software, LLC).

Cell viability via XTT assay. After desired aptamer labeling and subsequent incubation, the XTT reagent (2,3-bis-(2-methoxy-4-nitro-5-sulfophenyl)-2H-tetrazolium-5-carboxanilide, disodium salt, Biotium 10060) was activated by combining 8 µL of menadione stock (1.7 mg/mL in acetone, stored as aliquots at −80 °C) with 992 µL of XTT stock (1 mg/mL in PBS, stored as aliquots at –80 °C). Then, the activated XTT reagent was poured into a trough added (40 µL) to each well of the 96-well. Immediately after the addition of the activated XTT reagent, the absorbance at 450 nm and 630 nm was measured on a Tecan M1000 microplate reader, which served as the "initial" measurement. The plate was removed from the plate reader and incubated for 2 h at 37 °C. After 2 hours, the absorbance at 450 nm and 630 nm was measured again, which served as the "final" measurement.

Any background was removed from the data by subtracting each 630 nm "initial" measurement from the respective 450 nm "initial" measurement. This was repeated for the final measurements. The change in background-subtracted absorbance over the 2 h timeframe was calculated by subtracting each "initial" value from its respective "final" value. Therefore, each well should have the following equation performed: (Abs450 − Abs630)final − (Abs450 − Abs630)initial.

Then, the adjusted values were used to calculate the averages for the triplicate negative control data. The data was normalized the negative control such that the negative control was equal to 1. The data was presented and analyzed using Graphpad Prism v10.5.0 (GraphPad Software, LLC).

Supporting Figures and Tables

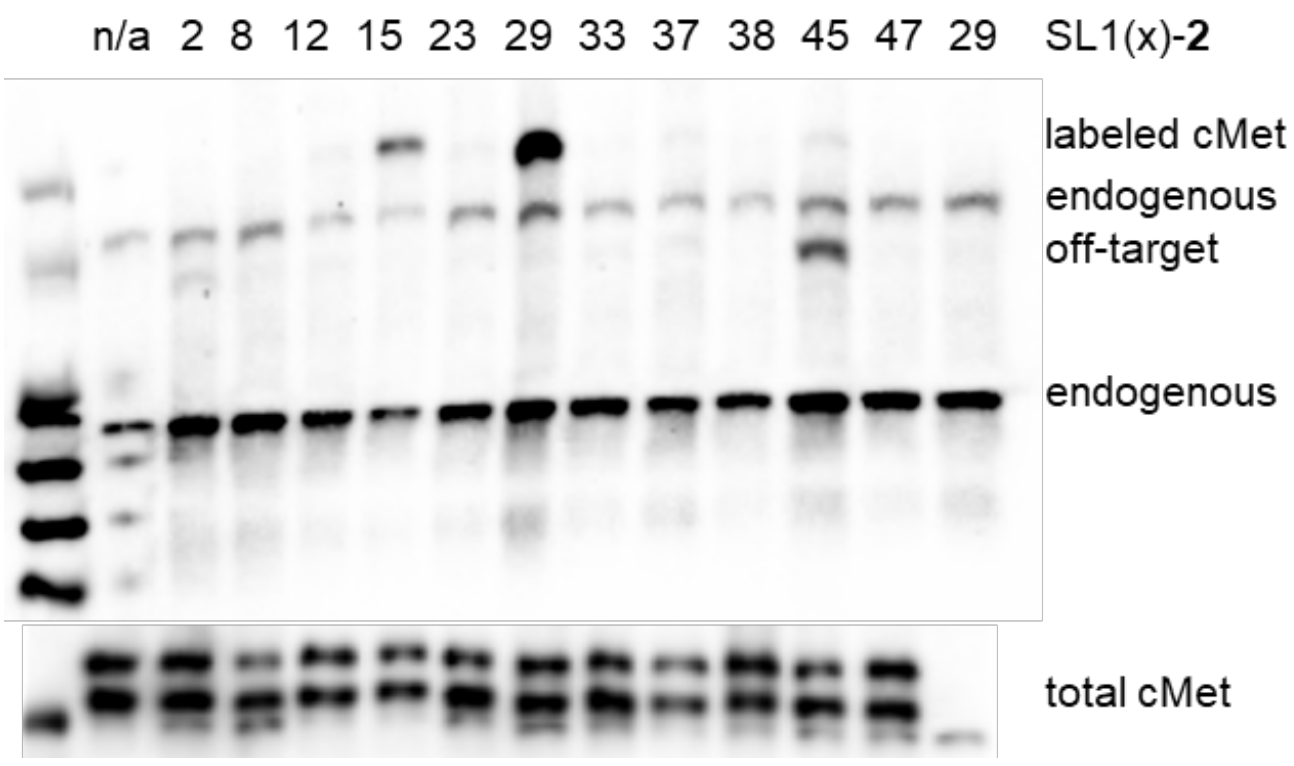

Figure S1. Full SA-HRP and anti-GFP western blot from **Figure 2B**.

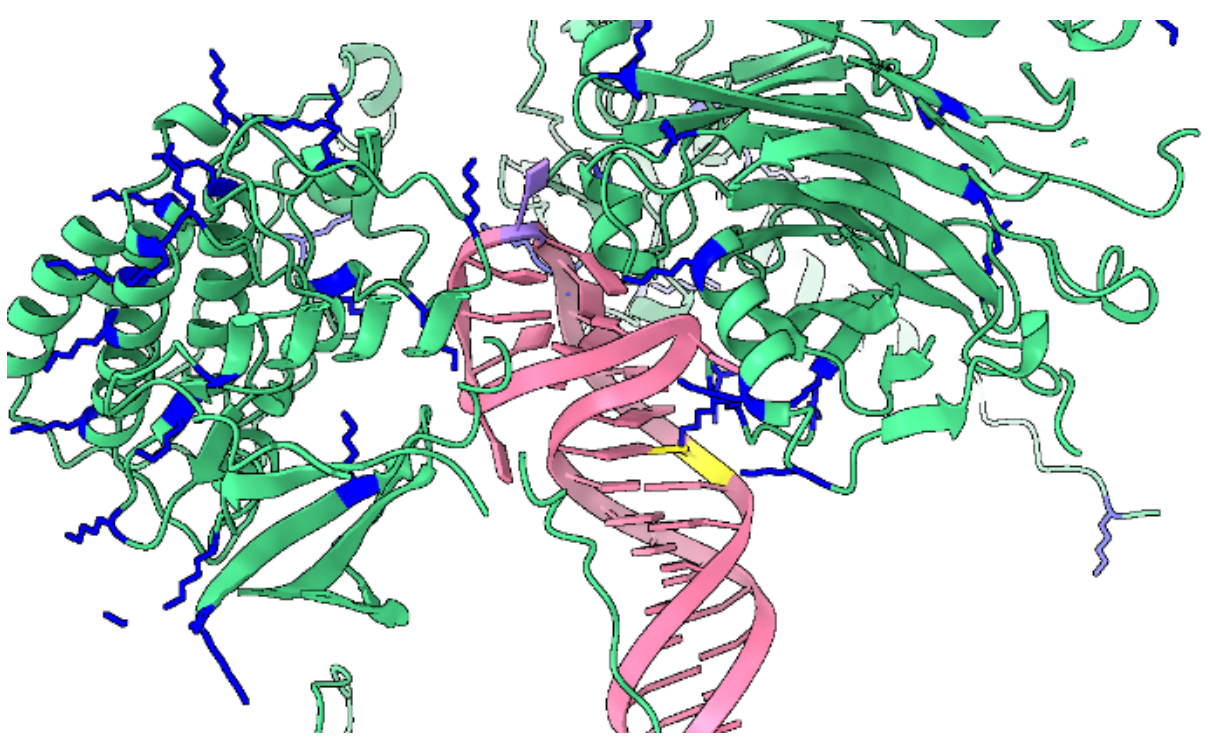

Figure S2. Predicted AlphaFold structure of SL1 (pink) binding to cMet (green). Lysine residues are indicated in blue. T29 is colored purple and T23 is colored yellow, showing position 29 for proper orientation.[5]

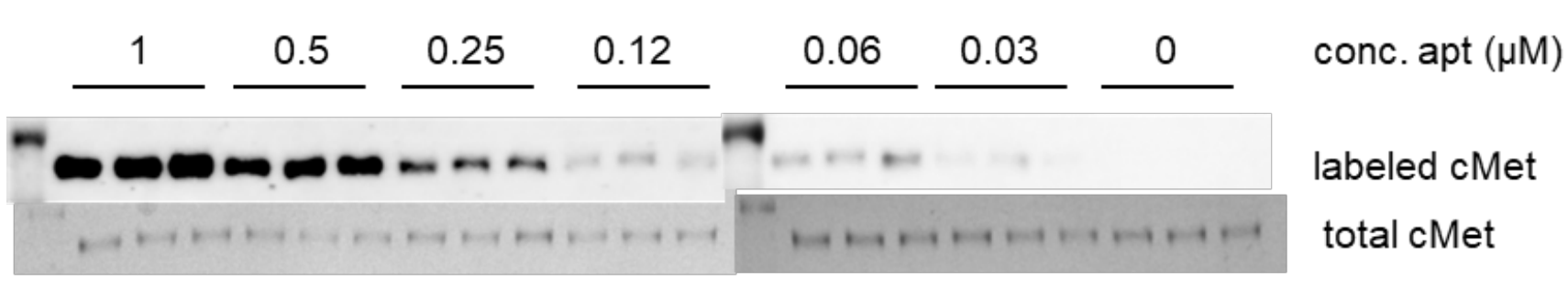

Figure S3. SA-HRP blot analysis of dose-dependent labeling of cMet (0.1 μM) with indicated concentrations of SL1(29)-**2** for 1 h at 37 °C in supplemented DPBS containing 0.1 mg/mL yeast tRNA. For quantification shown in **Figure 2D**, biotin band intensities were divided by the total protein, and then normalized to the highest intensity band (aptamer = 1 μM).

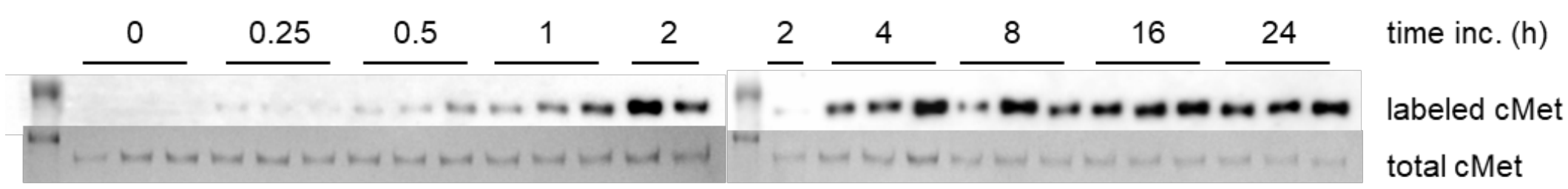

Figure S4. SA-HRP blot and silver stain analysis of time course labeling of cMet (0.1 µM) with SL1(29)-**2** (0.25 µM) for indicated amount of time at 37 °C in supplemented DPBS containing 0.1 mg/mL yeast tRNA. For quantification shown in **Figure 2F**, biotin band intensities were divided by the total protein, and then normalized to the highest intensity band (time = 24 h).

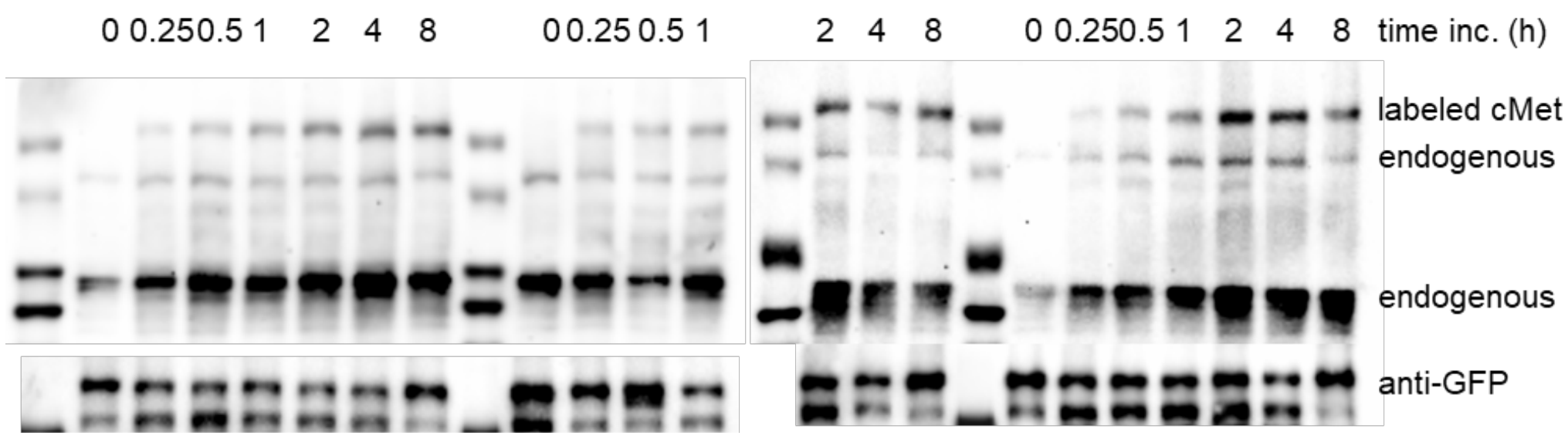

Figure S5. Full SA-HRP and anti-GFP blot analysis of time course labeling of HEK293T cells transfected with cMet-GFP. SL1(29)-**2** (0.5 µM) was incubated for the indicated amount of time at 37 °C in supplemented DPBS containing 0.1 mg/mL yeast tRNA. For quantification shown in **Figure 3C**, biotin band intensities were divided by the total protein, and then normalized to the highest intensity band (time = 8 h).

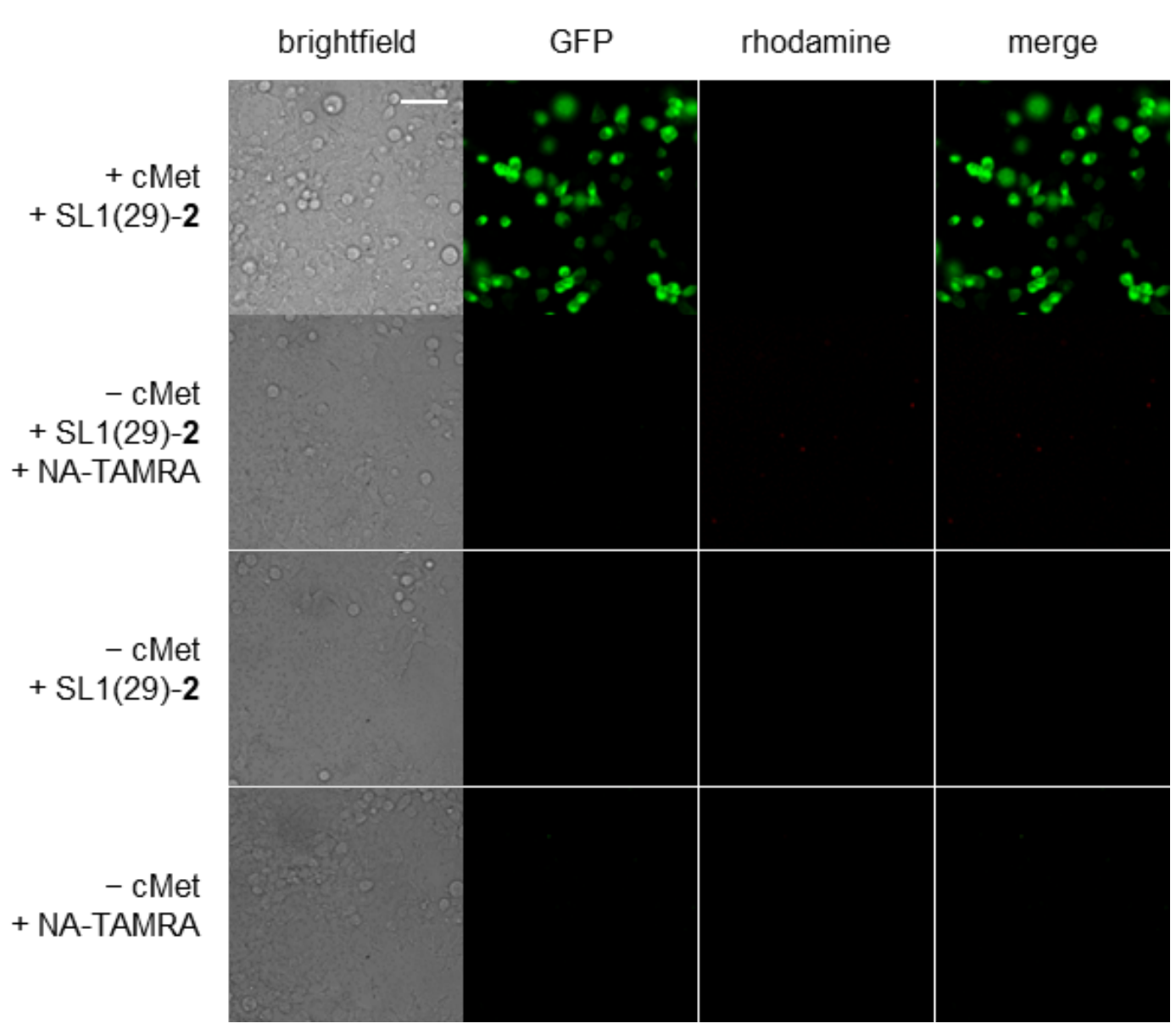

Figure S6. Fluorescence images of HEK cells expressing cMet-GFP or cMet negative HEK cells incubated with SL1(29)-**2** (0.5 µM) for 2 h and subsequently incubated with 10 µg/mL neutravidin tetramethylrhodamine (NA-TAMRA) for 5 minutes. Scale bar = 50 µm.

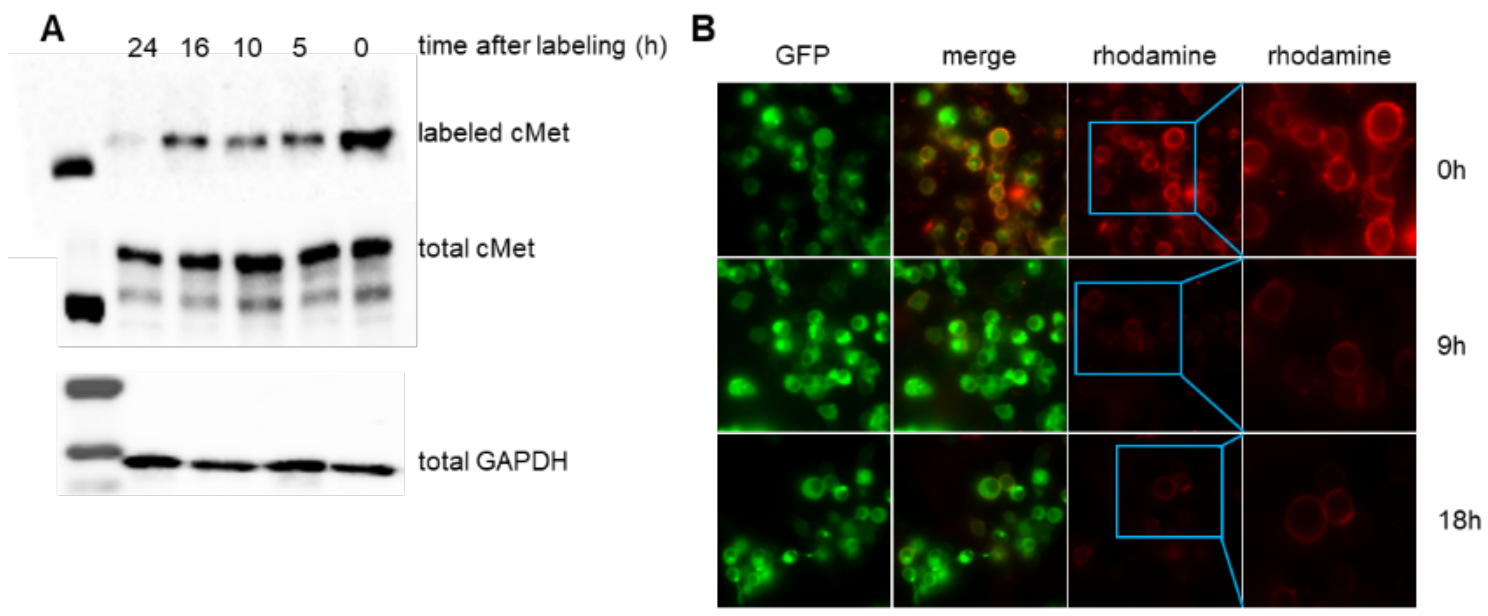

Figure S7. (A) cMet-GFP expressing HEK cells were incubated with SL1(29)-**2** (0.5 µM) for 1 hour at 37 °C in supplemented DPBS with 0.1 mg/mL tRNA, followed by one wash with supplemented DPBS. Cells were then incubated in DMEM with 10% FBS at 37 °C for the indicated amount of time. Cells were then analysis for duration of biotin label via SDS-PAGE. (B) cMet-GFP expressing HEK cells were SL1(29)-**2** (0.5 µM) for 1 hour at 37 °C in supplemented DPBS followed by one wash with supplemented DPBS. Cells were then incubated in DMEM with 10% FBS at 37 °C for the indicated time. Cells were then incubated with 10 µg/mL NA-TAMRA for 5 minutes and analyzed via fluorescence imaging for the presence of cell surface biotin.

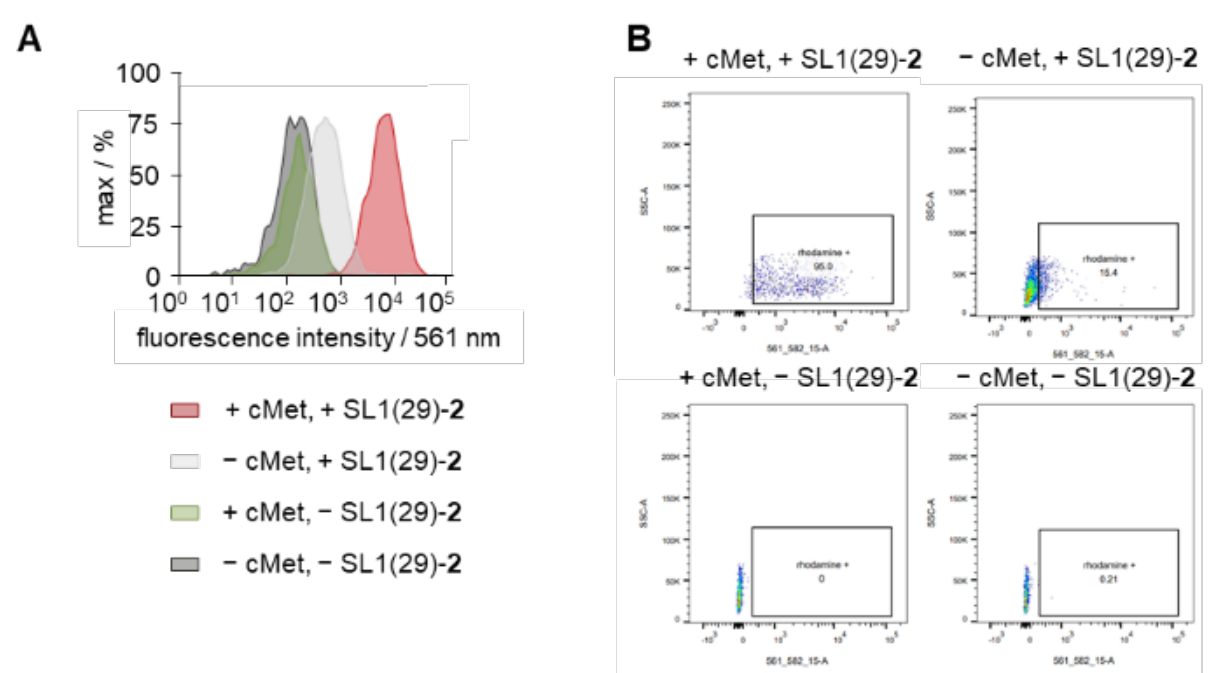

Figure S8. (A) Normalized histogram and (B) dot plots of flow cytometry analysis of HEK cells with or without cMet-GFP expression incubated with SL1(29)-**2** (0.5 µM) for 2 h and subsequently incubated with 10 µg/mL NA-TAMRA for 5 minutes. Cells were gates for GFP fluorescence before fluorescence intensity evaluation. 20,000 events were recorded.

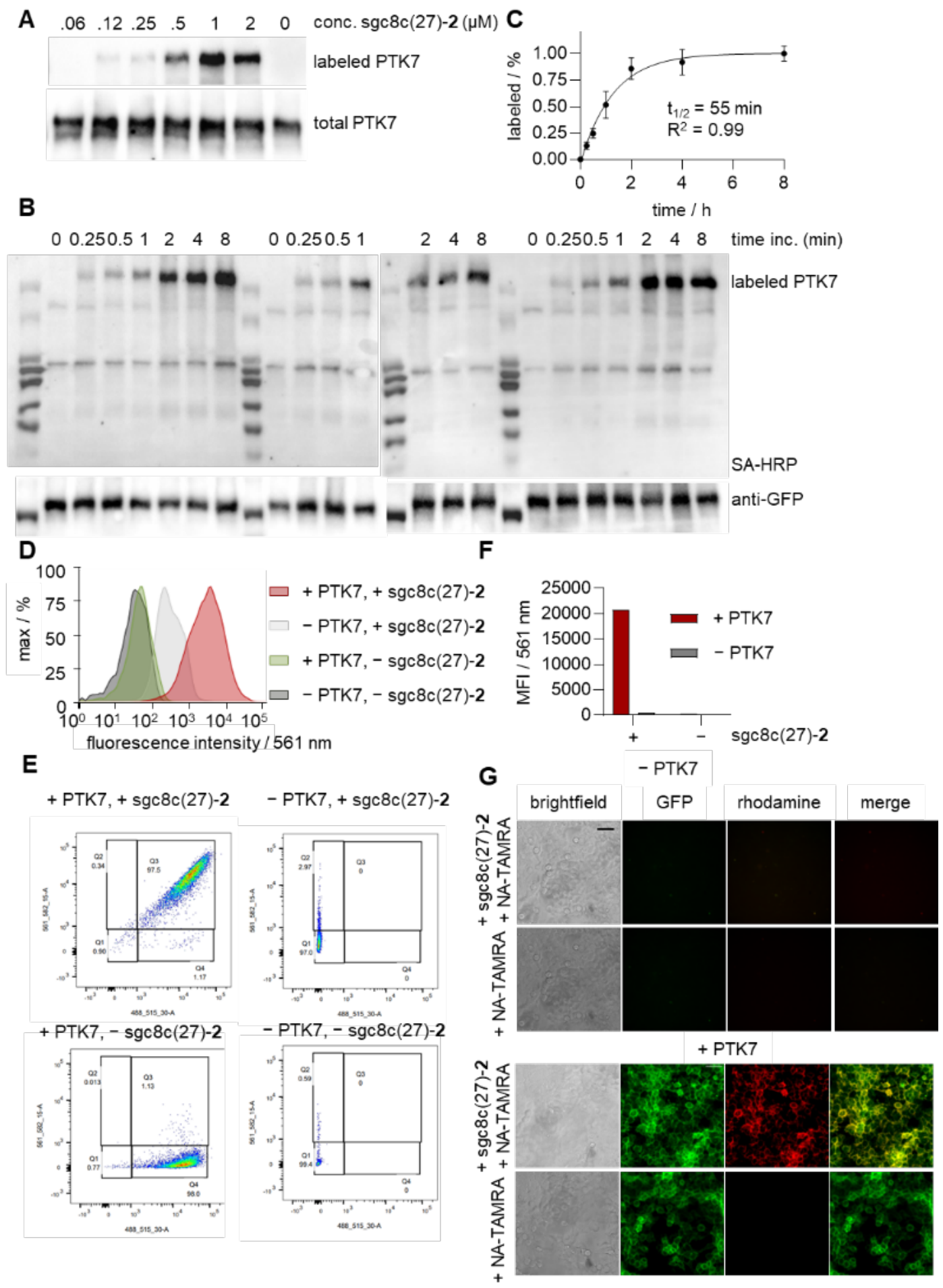

Figure S9. (A) SA-HRP and anti-GFP blot of HEK:PTK7-GFP cells incubated with the indicated concentration of sgc8c(27)-**2** for 2 h in supplemented DPBS containing 0.1 mg/mL tRNA at 37 ºC. (B) SA-HRP and anti-GFP blots and (C) quantification of HEK:PTK7-GFP cells incubated with sgc8c(27)-**2** (0.5 μM) for the indicated amount of time in supplemented DPBS containing 0.1 mg/mL tRNA at 37 ºC. Biotin band intensities were divided by the total protein and then normalized to the highest intensity band (time = 8 h). (D) Normalized histograms, (E) dot plots, (F) MFI, and (G) fluorescence imaging analysis of HEK:PTK7-GFP cells incubated with sgc8c(27)-**2** (0.5μM) for 2 h in supplemented DPBS containing 0.1 mg/mL tRNA at 37 ºC. For flow cytometry, 20,000 events were recorded. Scale bar = 50 μm.

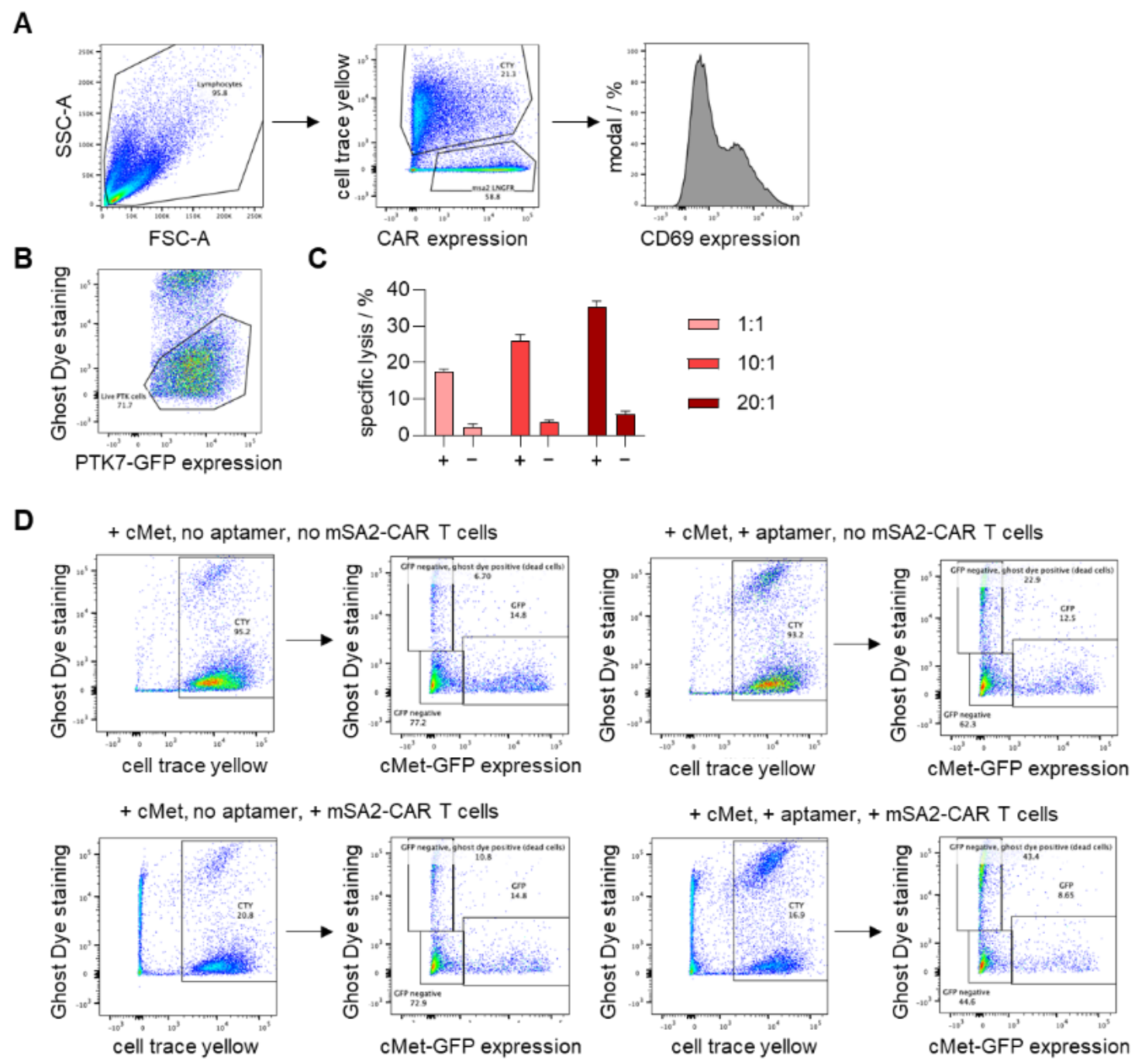

Figure S10. (A) Gating of CAR T cells for determination of CD69 expression (10:1 E:T ratio). (B) Gating of live (Ghost Dye negative) HEK:PTK7-GFP cells after co-incubation with mSA2-CAR T cells in **Figure 4C**. (C) Specific lysis of biotinylated HEK:PTK7-GFP co-incubated with different E:T ratios of mSA2-CAR T cells via ghost dye staining. Bars represent averages and error bars represent standard deviations from three independent experiments. (D) Gating of cMet-GFP transfected HEK cells after incubation with or without SL1(29)-**2** and/or mSA2-CAR T cells, as indicated. 20,000 events were recorded.

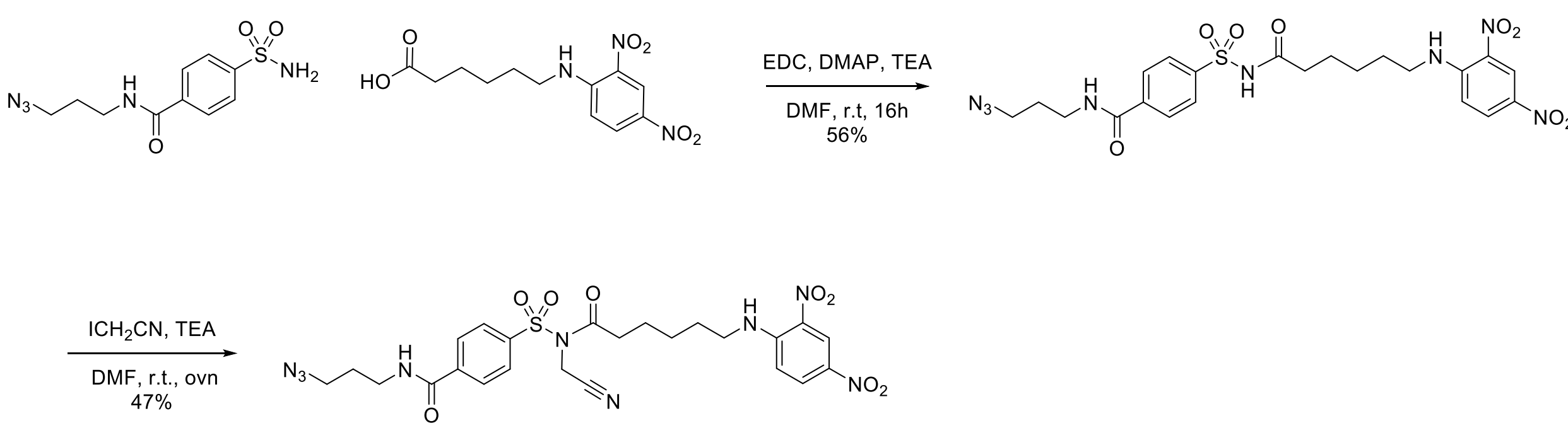

3

Figure S11. Chemical synthesis of DNP-transferring *N*-acyl sulfonamide electrophile **3**.

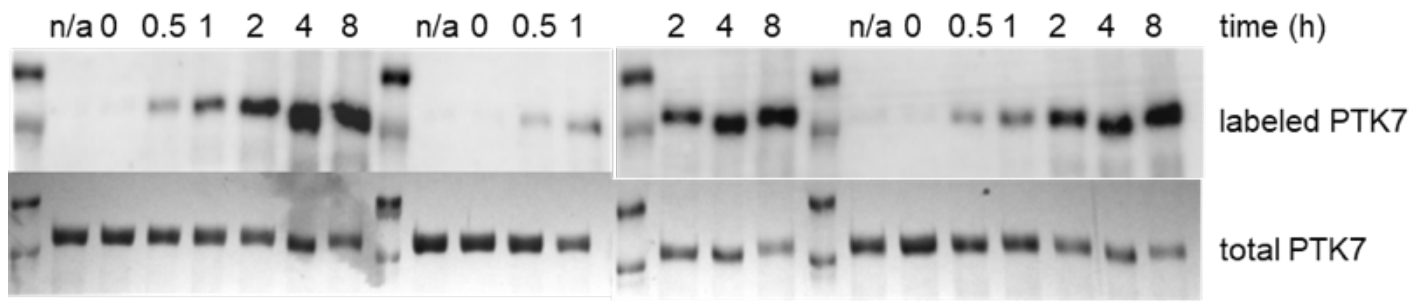

Figure S12. anti-DNP blot and silver stain analysis of time course labeling of PTK7 (0.1 μM) with sgc8c(27)-**3** (0.25 μM) for indicated amount of time at 37 ºC in supplemented DPBS containing 0.1 mg/mL yeast tRNA. For quantification shown in **Figure 5C**, DNP n band intensities were divided by the total protein and then normalized to the highest intensity band (time = 8h).

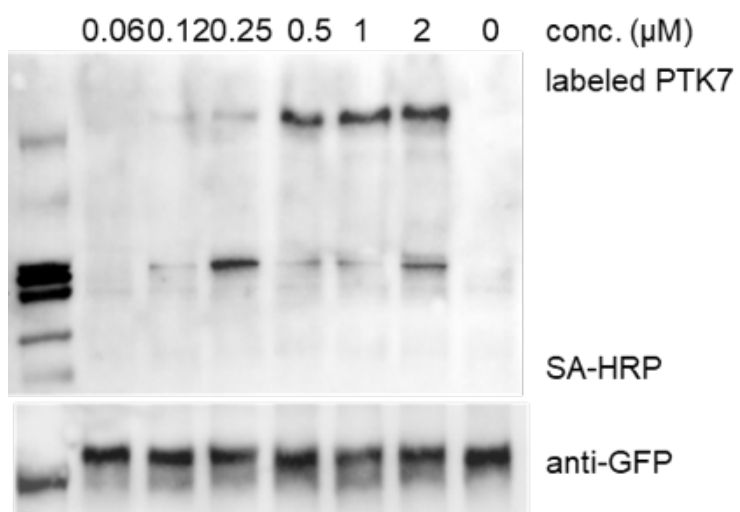

Figure S13. Full anti-DNP and anti-GFP blot of sgc8c(27)-**3** labeling of HEK:PTK7-GFP cells for 2 h in supplemented DPBS containing 0.1 mg/mL tRNA at 37 ºC.

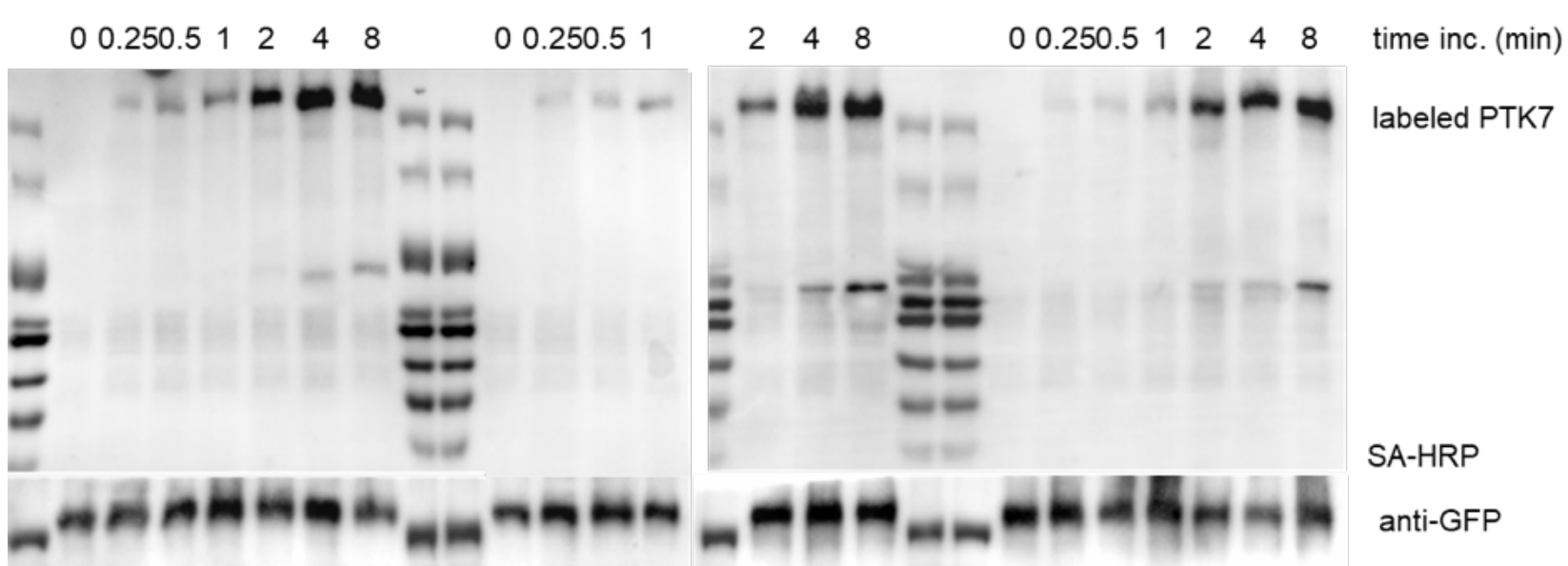

Figure S14. anti-DNP and anti-GFP blots analysis of HEK:PTK7-GFP cells incubated with sgc8c(27)-**3** (0.5 μM) for the indicated amount of time in supplemented DPBS containing 0.1 mg/mL tRNA at 37 ºC. For quantification shown in **Figure 5F,** DNP band intensities were divided by the total protein, and then normalized to the highest intensity band (time = 8 h).

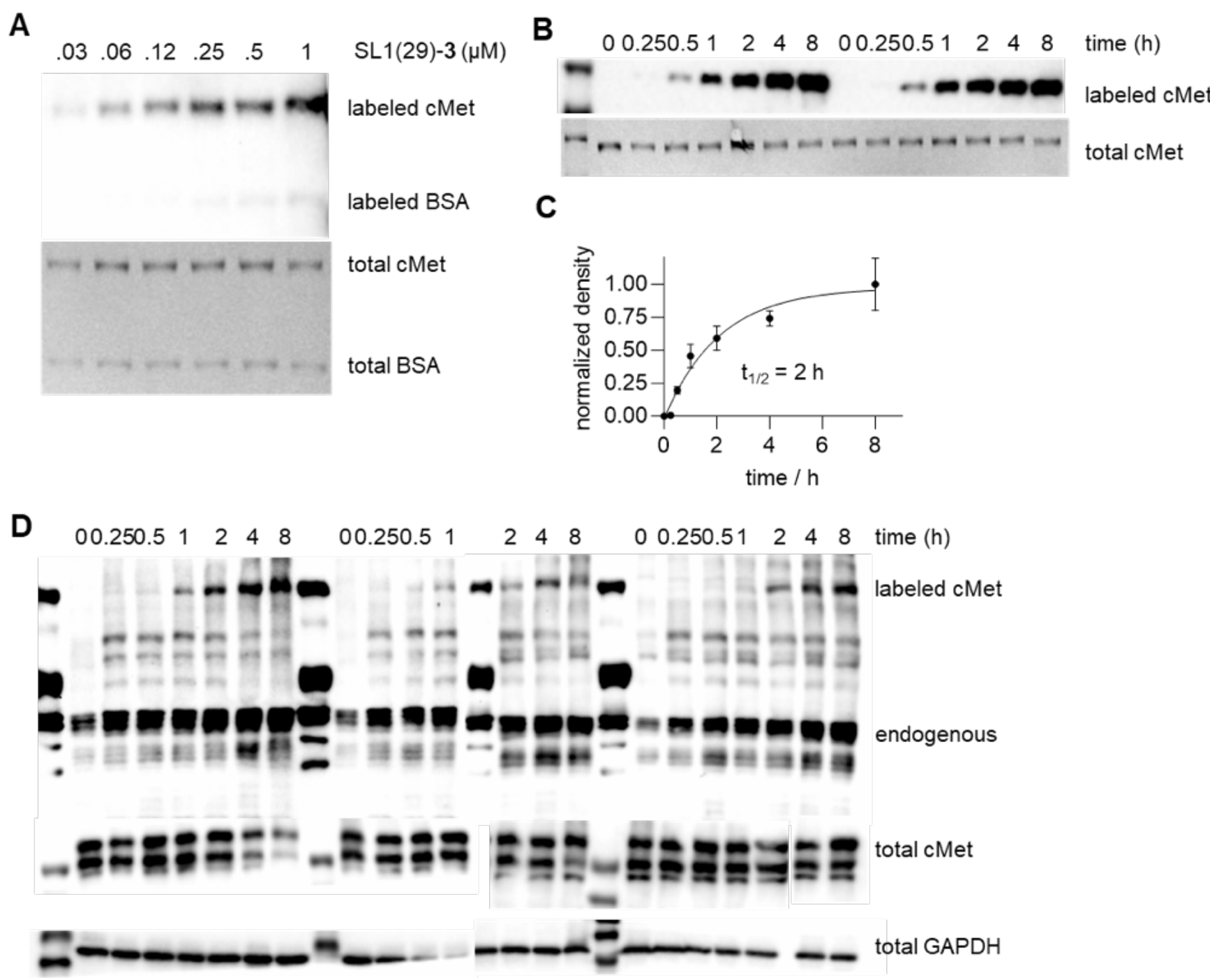

Figure S15. (A) Selectivity analysis of DNP transfer to cMet (0.1 μM) dependent on the concentration of SL1(29)-**3** in the presence of BSA (0.1 μM). (B) anti-DNP and anti-GFP blots and (C) quantification analysis of HEK cells transfected with cMet-GFP incubated with SL1(29)-**3** (0.5 μM) for the indicated amount of time in supplemented DPBS containing 0.1 mg/mL tRNA at 37 ºC. For quantification, DNP band intensities were divided by the total protein, and then normalized to the highest intensity band (time = 8 h).

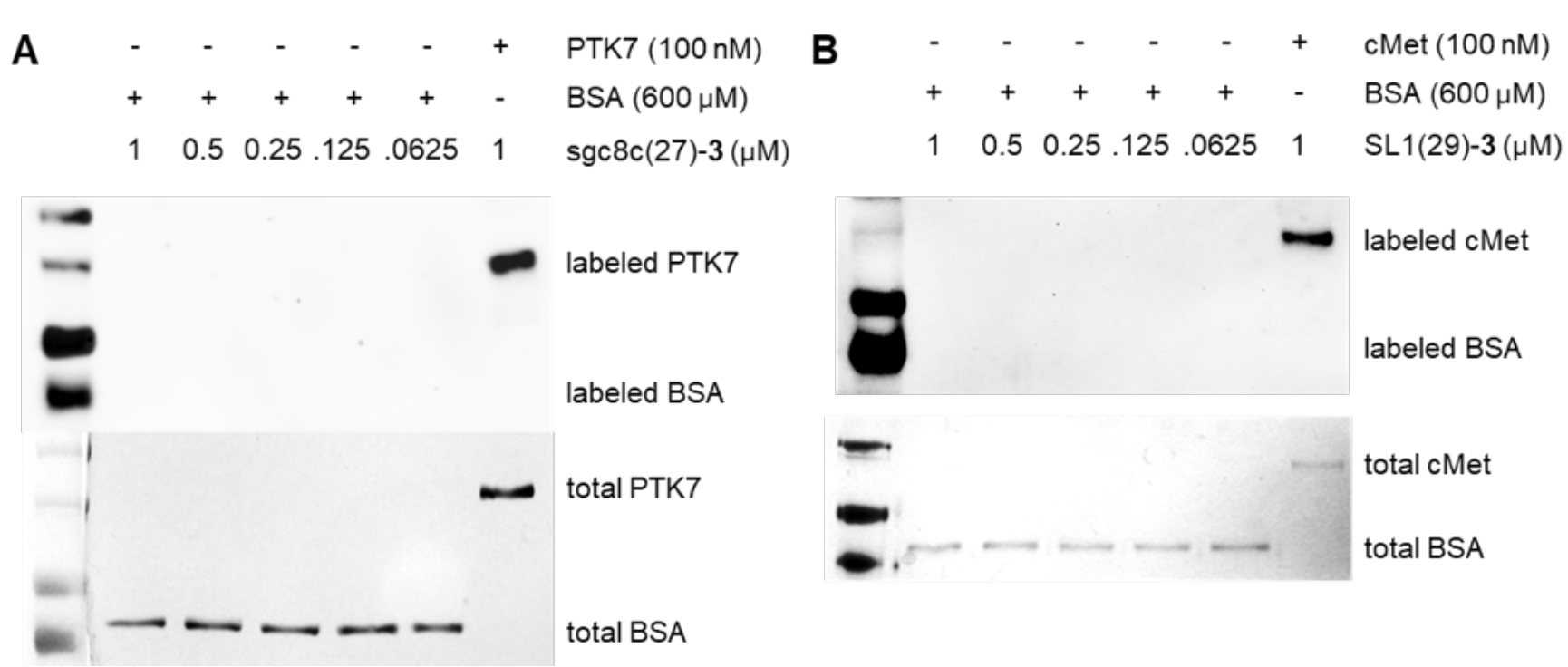

Figure S16. BSA (600 μM) was incubated with the indication concentration of either (A) sgc8c(27)-**3** or (B) SL1(29)-**3** for 2 hours in supplemented DPBS containing 0.1 mg/mL tRNA at 37 ºC. In parallel, recombinant PTK7 or cMet (100 nM) was incubated with sgc8c(27)-**3** or Sl1(29)-**3** (1 μM) respectively for 2 hours in supplemented DPBS. BSA labeling reactions were then diluted to 100 nM and analyzed alongside PTK7 for DNP modification by SDS-PAGE followed by silver staining and western blot analysis.

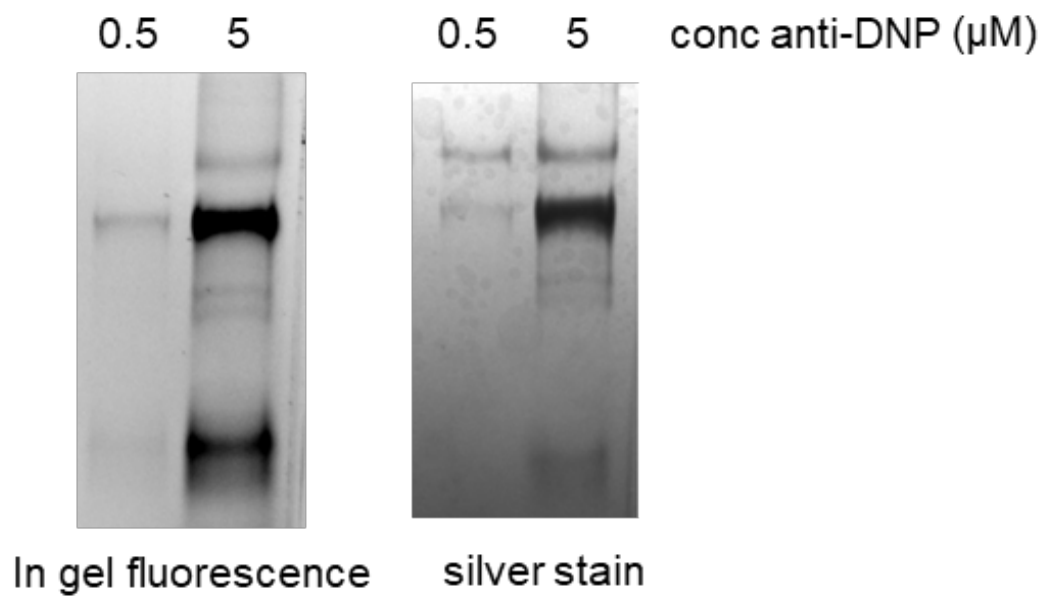

Figure S17. SDS-PAGE analysis of NHS-TAMRA labeled αDNP.

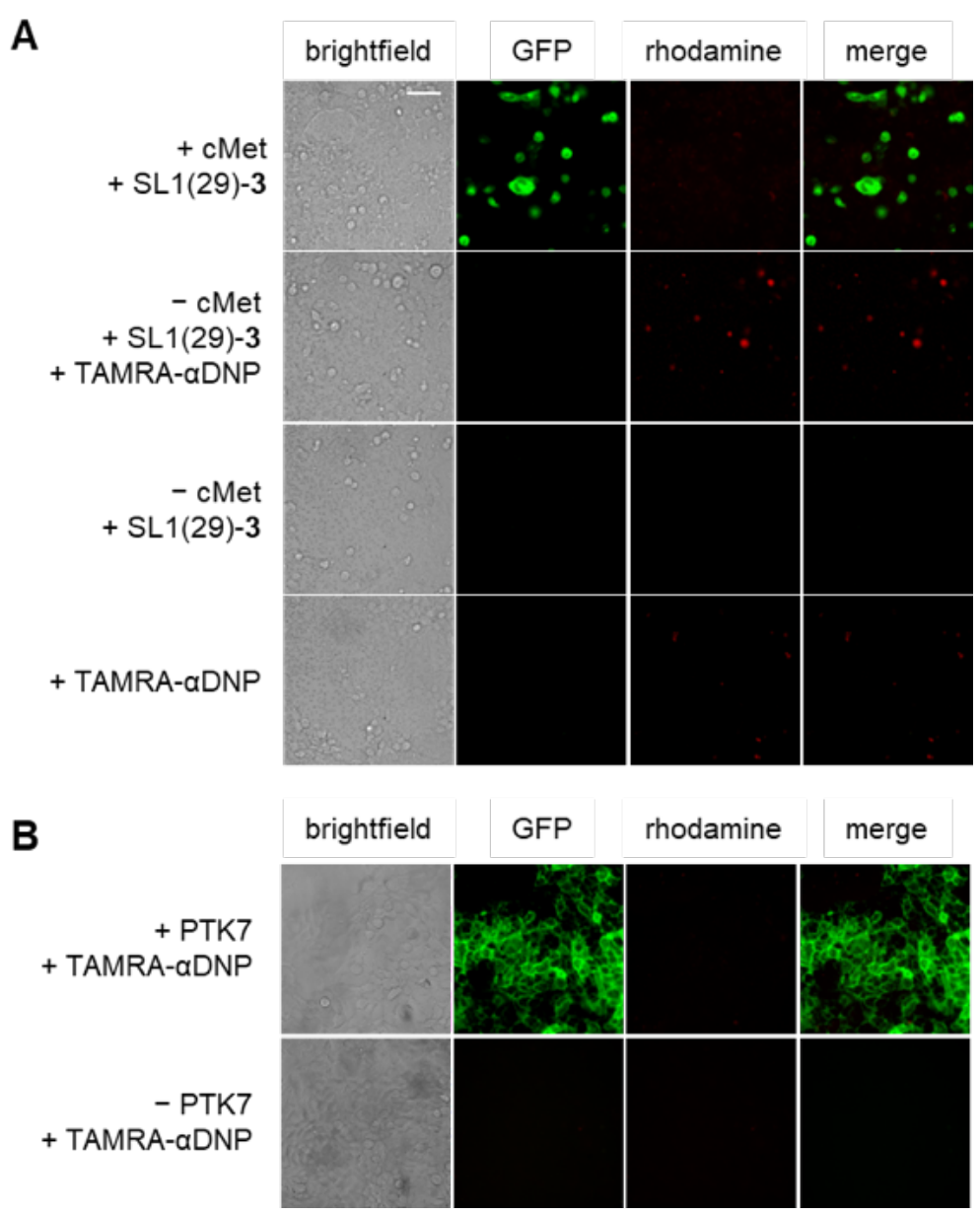

Figure S18. (A) Fluorescence images of HEK cells expressing cMet-GFP or cMet negative HEK cells incubated with SL1(29)-**3** (0.5 µM) for 2 h and subsequently incubated with 10 µg/mL TAMRA-αDNP for 20 minutes. (B) Fluorescence images of HEK:PTK7-GFP or PTK7 negative HEK cells incubated with TAMRA-αDNP for 20 minutes. Scale bar = 50 µm.

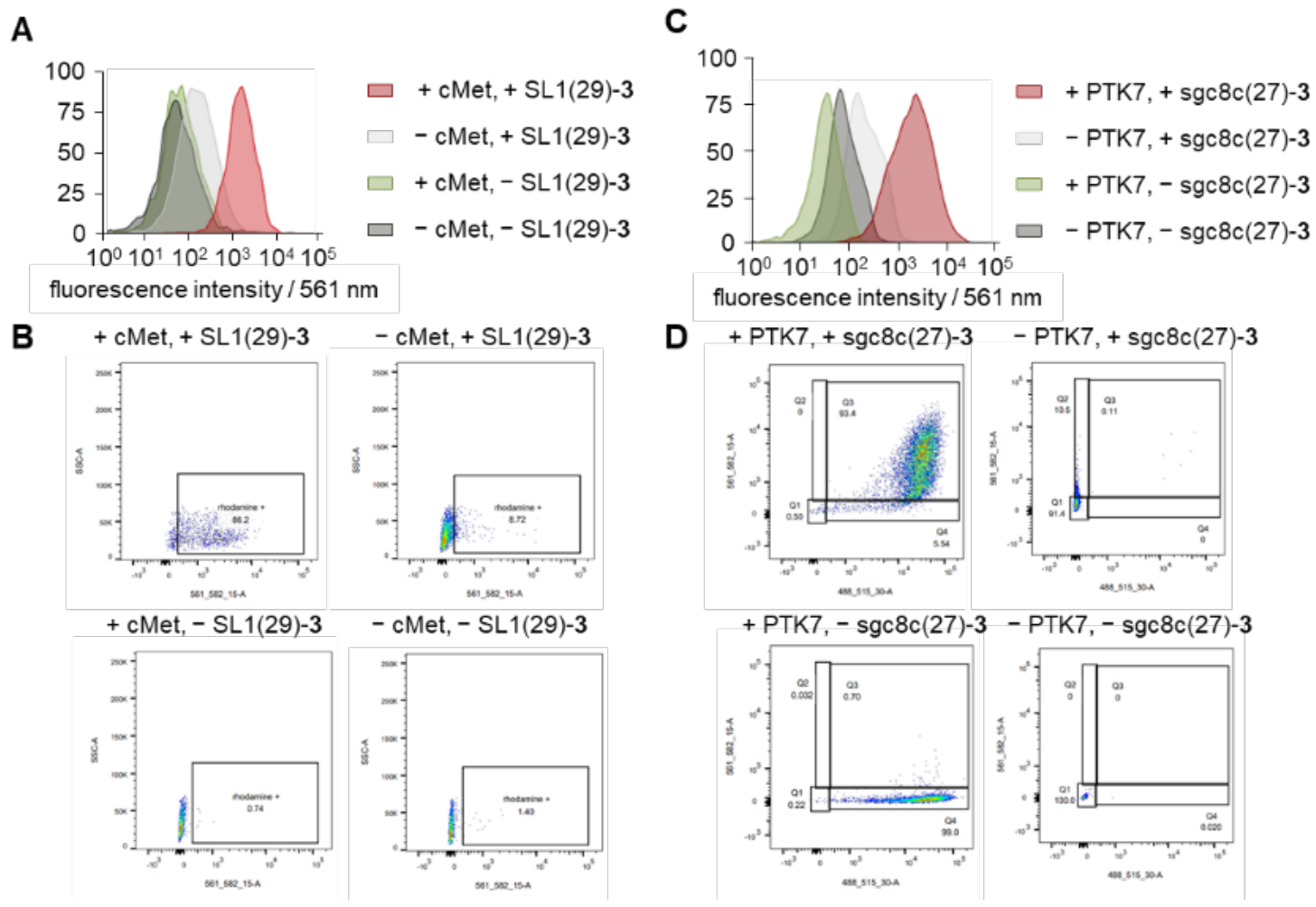

Figure S19. (A) Normalized histogram (B) dot plots of flow cytometry analysis of HEK cells with or without cMet-GFP expression incubated with SL1(29)-**3** (0.5 µM) for 2 h. (C) Normalized histogram and (D) dot plots of flow cytometry analysis of HEK cells PTK7-GFP expression incubated with sgc8c(27)-**3** (0.5 µM) and subsequently incubated with 10 µg/mL TAMRA-αDNP for 20 minutes. 20,000 events were recorded.

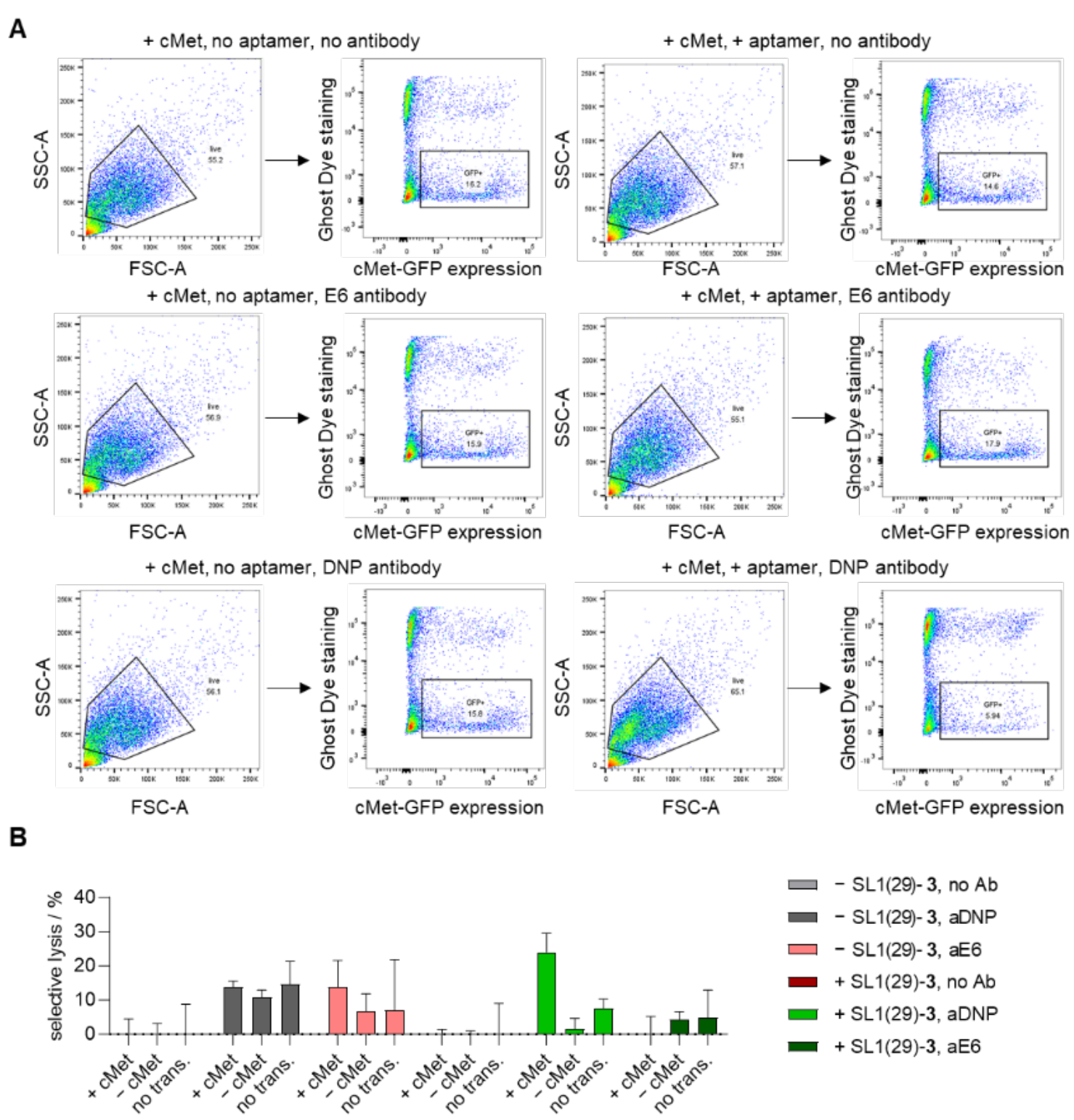

Figure S20. (A) Gating of cMet-GFP transfected HEK cells after incubation with or without SL1(29)-**3** and the indicated antibody in the presence of reconstituted complement serum, as indicated. 20,000 events were recorded. (B) Selective lysis of HEK cells with or without cMet-GFP expression incubated with SL1(29)-**3** (0.5 µM) for 2 h and incubated with 10% [v/v] baby rabbit complement and 50 µg/mL of the indicated antibody for 6 h, as detected by ghost dye staining.

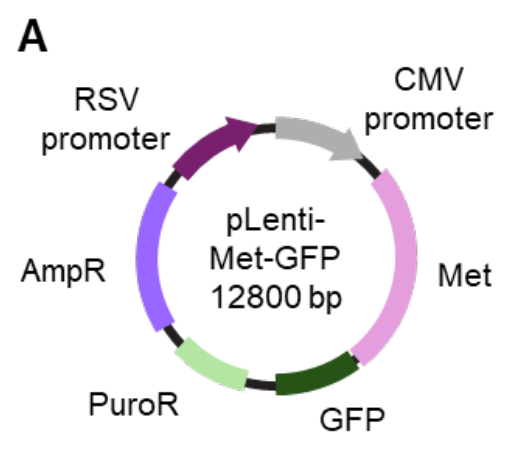

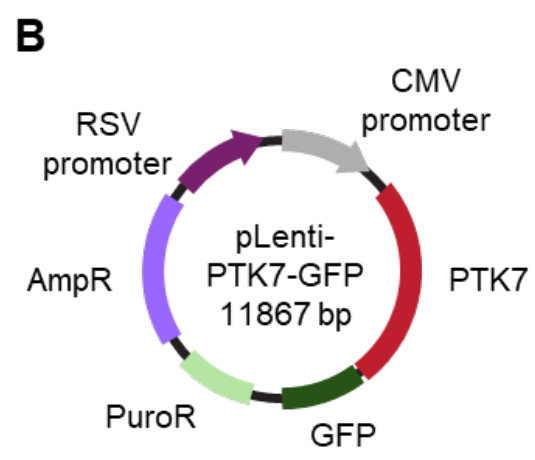

Figure S21. Plasmid maps of (A) pLenti-MetGFP and (B) pLenti-PTK7-GFP for mammalian expression and lentiviral production.

Table S1. Volumes for preparation of transfection reagents.

	**Tube A**			**Tube B**		**Total volume (µL/well)**
**plate size**	**OMEM (µL/well)**	**DNA (ng/well)**	**P3000 (µL/well)**	**OMEM (µL/well)**	**Lipofectamine 3000 (µL/well)**	
96-well	to 5	200	0.4	to 5	0.4	10
48-well	to 10	400	0.8	to 10	0.8	20
6-well	to 125	2000	4	to 125	4	250

Table S2. List of all modified aptamers used in this study.
The modified residue is highlighted and bolded.

**aptamer name**	**target protein**	**aptamer sequence**
SL1(2)-1	cMet	A**1**CAGGCTGGATGGTAGCTCGGTCGGGGTGGGTG GGTTGGCAAGTCTGAT
SL1(8)-1	cMet	ATCAGGC**1**GGATGGTAGCTCGGTCGGGGTGGGTG GGTTGGCAAGTCTGAT
SL1(12)-1	cMet	ATCAGGCTGGA**1**GGTAGCTCGGTCGGGGTGGGTG GGTTGGCAAGTCTGAT
SL1(15)-1	cMet	ATCAGGCTGGATGG**1**AGCTCGGTCGGGGTGGGTG GGTTGGCAAGTCTGAT
SL1(19)-1	cMet	ATCAGGCTGGATGGTAGC**1**CGGTCGGGGTGGGTG GGTTGGCAAGTCTGAT
SL1(23)-1	cMet	ATCAGGCTGGATGGTAGCTCGG**1**CGGGGTGGGTG GGTTGGCAAGTCTGAT

SL1(29)-1	cMet	ATCAGGCTGGATGGTAGCTCGGTCGGGG**1**GGGTGGGTTGGCAAGTCTGAT
SL1(33)-1	cMet	ATCAGGCTGGATGGTAGCTCGGTCGGGGTGGG**1**GGGTTGGCAAGTCTGAT
SL1(37)-1	cMet	ATCAGGCTGGATGGTAGCTCGGTCGGGGTGGGTGGG**1**TGGCAAGTCTGAT
SL1(38)-1	cMet	ATCAGGCTGGATGGTAGCTCGGTCGGGGTGGGTGGGT**1**GGCAAGTCTGAT
SL1(45)-1	cMet	ATCAGGCTGGATGGTAGCTCGGTCGGGGTGGGTGGGTTGGCAAG**1**CTGAT
SL1(47)-1	cMet	ATCAGGCTGGATGGTAGCTCGGTCGGGGTGGGTGGGTTGGCAAGTC**1**GAT
sgc8c(2)-1	PTK7	A**1**CTAACTGCTGCGCCGCCGGGAAAATACTGTACGGTTAGA
sgc8c(4)-1	PTK7	ATC**1**AACTGCTGCGCCGCCGGGAAAATACTGTACGGTTAGA
sgc8c(8)-1	PTK7	ATCTAAC**1**GCTGCGCCGCCGGGAAAATACTGTACGGTTAGA
sgc8c(11)-1	PTK7	ATCTAACTGC**1**GCGCCGCCGGGAAAATACTGTACGGTTAGA
sgc8c(27)-1	PTK7	ATCTAACTGCTGCGCCGCCGGGAAAA**1**ACTGTACGGTTAGA
sgc8c(30)-1	PTK7	ATCTAACTGCTGCGCCGCCGGGAAAATAC**1**GTACGGTTAGA
sgc8c(32)-1	PTK7	ATCTAACTGCTGCGCCGCCGGGAAAATACTG**1**ACGGTTAGA
sgc8c(37)-1	PTK7	ATCTAACTGCTGCGCCGCCGGGAAAATACTGTACGG**1**TAGA
sgc8c(38)-1	PTK7	ATCTAACTGCTGCGCCGCCGGGAAAATACTGTACGGT**1**AGA

Table S3. List of primers used in plasmid construction.

Primer	Fwd/Rev	Sequence (5' to 3')
P1	Fwd	ATAGAAGACACCGACTCTAGAGGATCCACCATGGGAGCTGCGCGGGGATC
P2	Rev	ATCCAGAGGTTGATTGTCGACGCGGCCGCTTACTTGTACAGCTCGTCCATGCCG
P3	Fwd	AGCGGCCGCGTCGACAATC
P4	Rev	GGTGGATCCTCTAGAGTCGGTGTCTTCTATGG

NMR Spectra

CR-4-72 1H 300 MHz DMSO

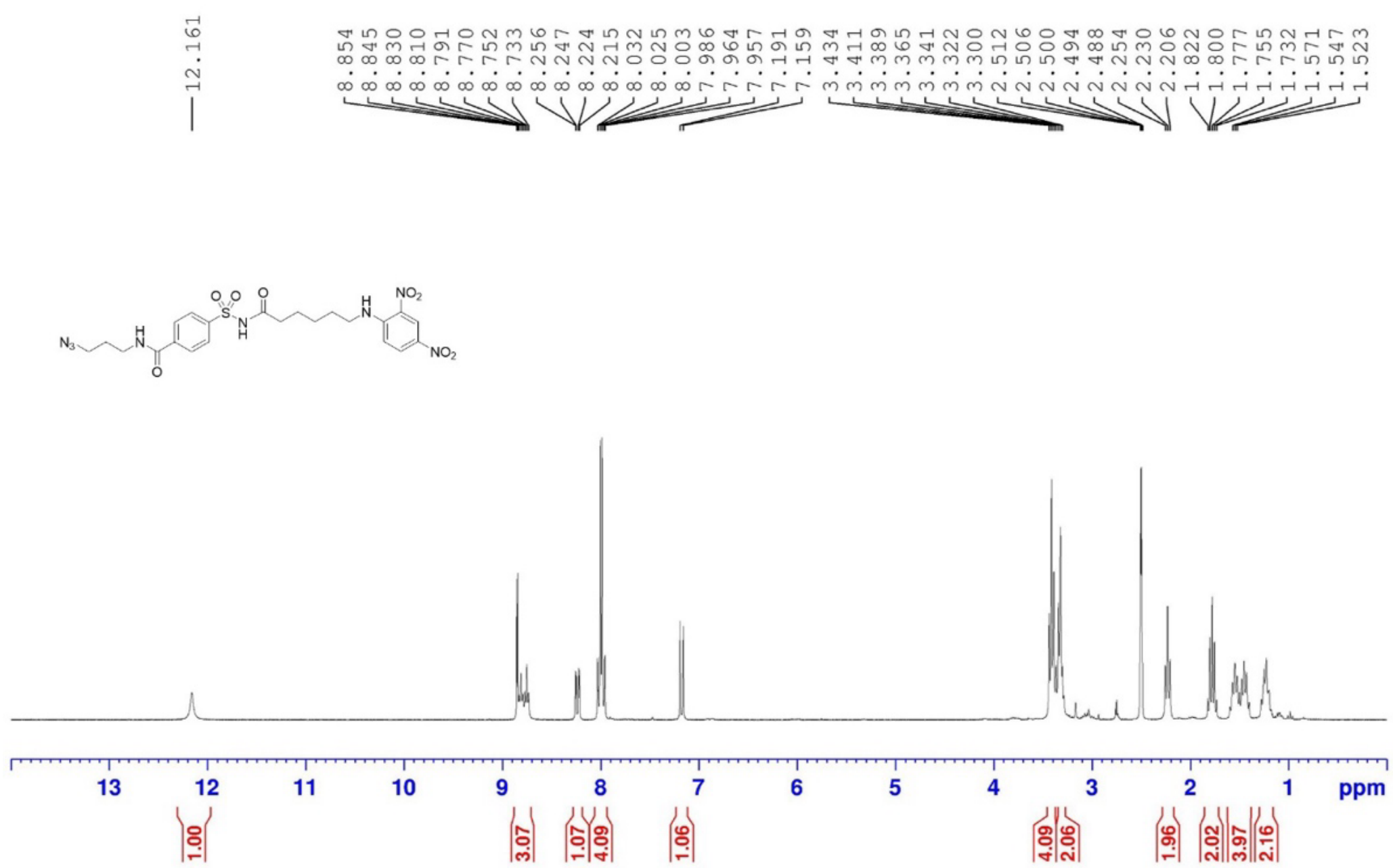

CR-4-72 13H 500 MHz dmso

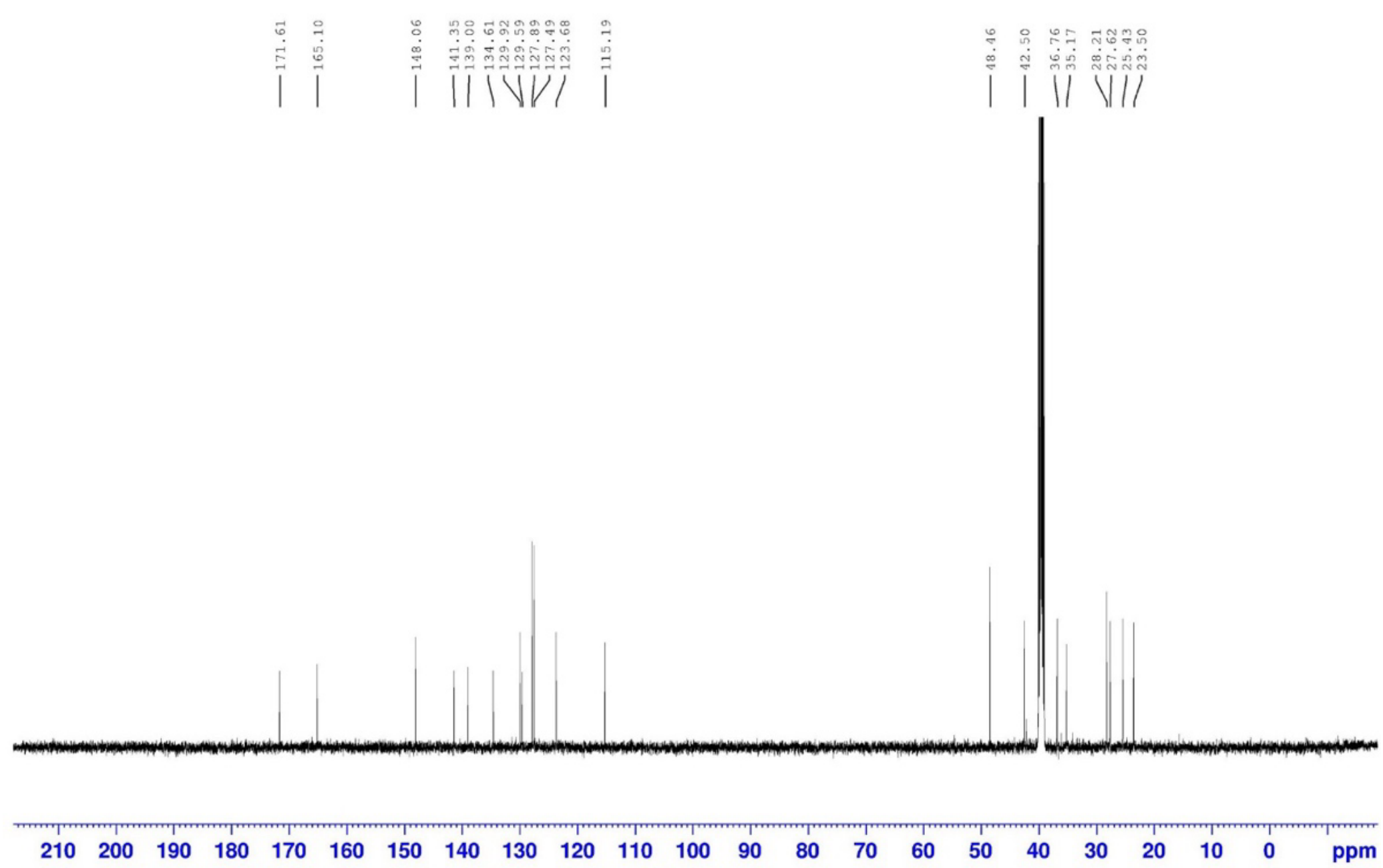

CR-4-74 1H 500 MHz dmso

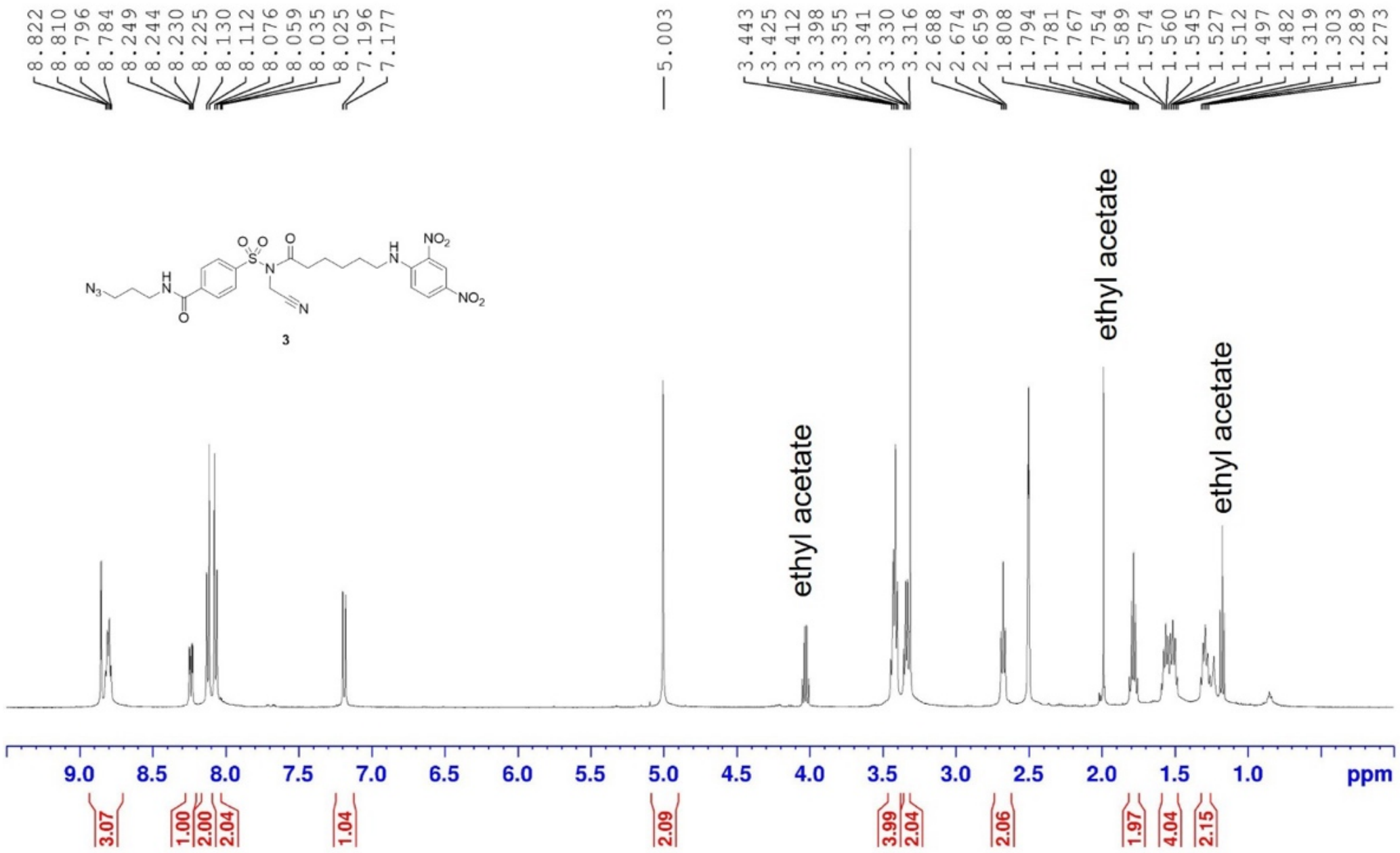

CR-4-74 1H3C 500 MHz dmso

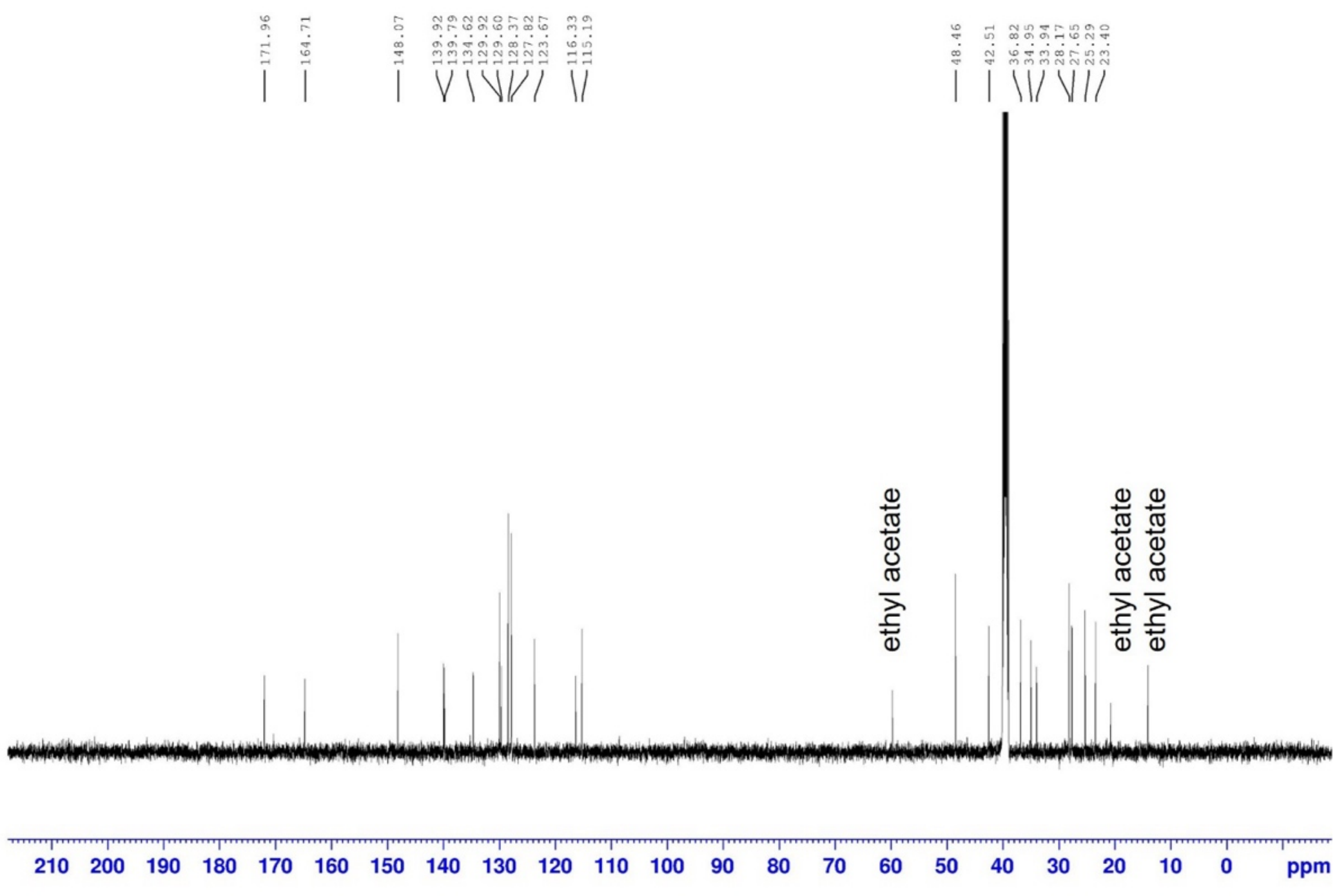

References

(1) Tivon, Y.; Falcone, G.; Deiters, A. Protein Labeling and Crosslinking by Covalent Aptamers. *Angew Chem Int Ed Engl* **2021**, *60* (29), 15899–15904. DOI: 10.1002/anie.202101174.

(2) Albright, S.; Cacace, M.; Tivon, Y.; Deiters, A. Cell Surface Labeling and Detection of Protein Tyrosine Kinase 7 via Covalent Aptamers. *J Am Chem Soc* **2023**, *145* (30), 16458–16463. DOI: 10.1021/jacs.3c02752.

(3) Gibson, D. G.; Young, L.; Chuang, R. Y.; Venter, J. C.; Hutchison, C. A., 3rd; Smith, H. O. Enzymatic assembly of DNA molecules up to several hundred kilobases. *Nat Methods* **2009**, *6* (5), 343–345. DOI: 10.1038/nmeth.1318.

(4) Kapcan, E.; Krygier, K.; da Luz, M.; Serniuck, N. J.; Zhang, A.; Bramson, J.; Rullo, A. F. Mimicry of molecular glues using dual covalent chimeras. *Nat Commun* **2025**, *16* (1), 2855. DOI: 10.1038/s41467-025-58083-z.

(5) Abramson, J.; Adler, J.; Dunger, J.; Evans, R.; Green, T.; Pritzel, A.; Ronneberger, O.; Willmore, L.; Ballard, A. J.; Bambrick, J.; et al. Accurate structure prediction of biomolecular interactions with AlphaFold 3. *Nature* **2024**, *630* (8016), 493–500. DOI: 10.1038/s41586-024-07487-w.
